# Plasticity of Fission Yeast CENP-A Chromatin Driven by Relative Levels of Histone H3 and H4

**DOI:** 10.1371/journal.pgen.0030121

**Published:** 2007-07-27

**Authors:** Araceli G Castillo, Barbara G Mellone, Janet F Partridge, William Richardson, Georgina L Hamilton, Robin C Allshire, Alison L Pidoux

**Affiliations:** Wellcome Trust Centre for Cell Biology, University of Edinburgh, Edinburgh, United Kingdom; European Molecular Biology Laboratory, Germany

## Abstract

The histone H3 variant CENP-A assembles into chromatin exclusively at centromeres. The process of CENP-A chromatin assembly is epigenetically regulated. Fission yeast centromeres are composed of a central kinetochore domain on which CENP-A chromatin is assembled, and this is flanked by heterochromatin. Marker genes are silenced when placed within kinetochore or heterochromatin domains. It is not known if fission yeast CENP-A^Cnp1^ chromatin is confined to specific sequences or whether histone H3 is actively excluded. Here, we show that fission yeast CENP-A^Cnp1^ can assemble on noncentromeric DNA when it is inserted within the central kinetochore domain, suggesting that in fission yeast CENP-A^Cnp1^ chromatin assembly is driven by the context of a sequence rather than the underlying DNA sequence itself. Silencing in the central domain is correlated with the amount of CENP-A^Cnp1^ associated with the marker gene and is also affected by the relative level of histone H3. Our analyses indicate that kinetochore integrity is dependent on maintaining the normal ratio of H3 and H4. Excess H3 competes with CENP-A^Cnp1^ for assembly into central domain chromatin, resulting in less CENP-A^Cnp1^ and other kinetochore proteins at centromeres causing defective kinetochore function, which is manifest as aberrant mitotic chromosome segregation. Alterations in the levels of H3 relative to H4 and CENP-A^Cnp1^ influence the extent of DNA at centromeres that is packaged in CENP-A^Cnp1^ chromatin and the composition of this chromatin. Thus, CENP-A^Cnp1^ chromatin assembly in fission yeast exhibits plasticity with respect to the underlying sequences and is sensitive to the levels of CENP-A^Cnp1^ and other core histones.

## Author Summary

The DNA of all genomes is organized into chromosomes that are packaged in chromatin in which DNA is wrapped around nucleosomes composed of the histones H2A, H2B, H3, and H4. Centromeres are the specialized regions on chromosomes that attach them to spindle microtubules, and this process is required to allow each daughter cell to receive one copy of each chromosome after they have duplicated. Centromere regions are distinguished from other parts of the chromosome by the incorporation of the histone H3 variant CENP-A instead of histone H3 into specialized nucleosomes. This CENP-A chromatin allows the machinery (the kinetochore) responsible for attaching chromosomes to microtubules to assemble. Fission yeast centromeres contain a central domain where CENP-A is prevalent. This study shows that CENP-A can associate with “foreign” DNA placed in the central domain. Therefore, CENP-A appears to associate with any DNA placed in this environment, independent of its DNA sequence. Increasing the relative level of H3 allows H3 to be assembled in place of CENP-A in this critical central domain and results in defective centromere/kinetochore function and chromosome segregation. This study highlights the plasticity of centromeric chromatin.

## Introduction

In most eukaryotes, chromosomes contain a centromere that occupies a single locus. The centromere acts as the site for assembly of the kinetochore that mediates the attachment of chromosomes to spindle microtubules and orchestrates their equational segregation to daughter nuclei at mitosis. In many organisms, long tandem arrays of repetitive satellite DNA, such as alpha-satellite DNA in humans, are found at each centromere [1,2].

Chromosomal DNA is packaged in chromatin composed of nucleosomes containing the four core histones H2A, H2B, H3, and H4. Histone variants can play specific roles in the regulation of gene expression. For example, H3.3 replaces H3 in regions of active transcription [3–5]. Intriguingly, kinetochores contain a specific form of chromatin in which canonical histone H3 is replaced by the centromere-specific histone H3 variant known generally as CENP-A [1,2,6,7].

CENP-A is essential for the assembly of a functional kinetochore and as such must represent a key component in establishing and/or maintaining the site of kinetochore assembly at the centromere [8–12]. Although CENP-A proteins are found at centromeres in all organisms, there appears to be no specific conserved sequence that ensures the assembly of CENP-A chromatin [1,2,7,13]. Indeed, the deposition of CENP-A appears to be malleable since inactivated human centromeres lack CENP-A, even though they retain alpha-satellite repeats [14]. In addition, neocentromeres occasionally arise on chromosomal DNA that lacks any similarity to alpha-satellite repeats, and CENP-A can associate with noncentromeric sequences included in human artificial chromosomes [14–19]. Similarly, in *Drosophila,* noncentromeric DNA can acquire the ability to assemble and propagate kinetochore proteins [20–22]. These and other observations suggest that the site of CENP-A chromatin assembly is epigenetically regulated and propagated [1,2,13].

**Figure 1:**
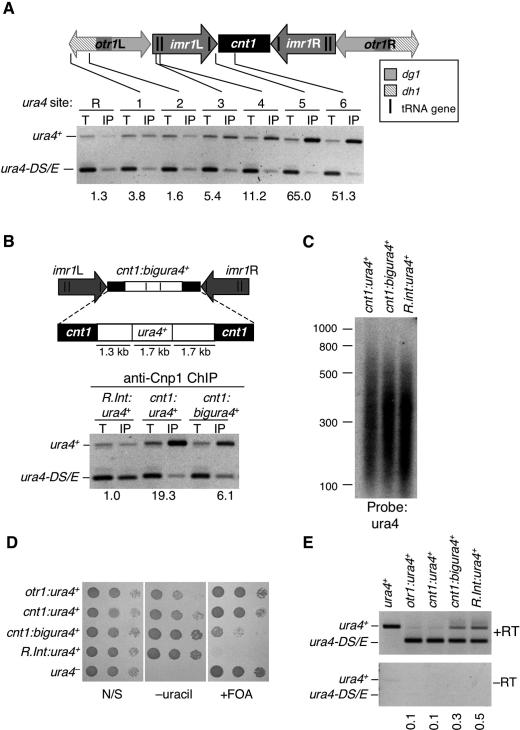
CENP-A^Cnp1^ Chromatin Can Associate with Noncentromeric DNA and Silence Genes Inserted in the Central Domain (A) ChIP analysis of CENP-A^Cnp1^. CENP-A^Cnp1^ association with *ura4* ^+^ insertions in *otr1* (sites 1 and 2), *imr1* (sites 3 and 4), *cnt1* (sites 5 and 6), and *R.int.cnt1:ura4* ^+^ (site R). Enrichment of *ura4* ^+^ was assessed compared to *ura4-DS/E* in IP relative to total (T) extract. Site 1 corresponds to site 3 in Partridge et al. [36]; site 2 = site 4; site 3 = site 6; site 5 = site 8; site 6 = site 9. (B) ChIP analysis of CENP-A^Cnp1^ association with *cnt1:ura4* ^+^ and *cnt1:bigura4* ^+^ insertions at site 6 and *R.int.cnt1:ura4* ^+^ (site R). (C) Southern analysis of DNA fragments extracted from cross-linked chromatin utilized for ChIP in (B); hybridization with *ura4* ^+^ probe. (D) Serial dilution of indicated strains on N/S (nonselective), −uracil (lacking uracil), media with FOA. (E) RT-PCR of *ura4* mRNA relative to *ura4-DS/E* in the indicated strains.

Fission yeast centromeres are reminiscent of those of metazoa in that they contain repetitive elements (outer repeats, *otr*) that flank the central domain (see Figure 1). The central domain is composed of inner repeats *(imr)* surrounding the central core *(cnt)* [23–25]. Noncoding transcripts arising from the outer repeat provide a substrate for RNA interference (RNAi) that directs methylation of histone H3 on lysine 9 and the assembly of silent chromatin (reviewed in [26]). Within the central domain most histone H3 is replaced by the centromere-specific H3 variant CENP-A^Cnp1^ to form the unusual chromatin that occupies most of the 10–12 kb comprising *imr* and *cnt* [11,23,27–29]. Consistent with the notion that CENP-A^Cnp1^ chromatin is a signature of kinetochore activity, kinetochore-specific proteins are confined to the central domain [11,29–35]. At each centromere a cluster of tRNA genes demarcates the two distinct chromatin domains: outer repeat silent heterochromatin and kinetochore chromatin [23,36]. Deletion of one of the two tRNA genes from one side of a centromere allows heterochromatin to infiltrate the central domain [37]. The fact that all three fission yeast centromeres have a common organization of DNA elements suggests that the assembly of heterochromatin and CENP-A^Cnp1^ chromatin domains could be strictly governed by sequence.

Marker genes placed within outer repeat chromatin are silenced, and this requires RNAi components, Clr4 histone H3K9 methyltransferase and Swi6 (homologue of HP1) [36,38]. Genes are also silenced in the central domain, but this silencing is strongly dependent on kinetochore integrity rather than RNAi-mediated heterochromatin [31,35,36,39]. Mutations in several kinetochore proteins, including CENP-A^Cnp1^, alleviate silencing, specifically in the central domain, and affect the association of CENP-A^Cnp1^ with the central domain and/or the unusual chromatin found within the central domain [11,29,35,36].

Dissection of fission yeast centromeric DNA showed that outer repeat and central domain sequences are required for the assembly of a kinetochore that supports mitotic segregation [23,24,40]. In *Drosophila* and humans, centromeres can arise at sites apparently lacking particular features at the primary DNA sequence level. It is not known whether the similarity between fission yeast and metazoan centromeres extends to the ability of CENP-A chromatin to assemble on noncentromeric sequences or if CENP-A assembly in this organism more closely resembles the situation in budding yeast in which CENP-A associates at a specific sequence.

Here, we investigate the ability of fission yeast CENP-A^Cnp1^ chromatin to assemble on noncentromeric sequences. We determine whether the amount of CENP-A^Cnp1^ incorporation directly correlates with silencing, kinetochore assembly, and kinetochore function. Altering the relative ratios between H3, H4, and CENP-A^Cnp1^ influences the assembly of CENP-A^Cnp1^ chromatin, the recruitment of other kinetochore proteins, and the fidelity of chromosome segregation. Surprisingly, there is no impediment to depositing H3 in the central domain. Thus, our observations indicate that the relative levels of histones are crucial for the correct formation of CENP-A^Cnp1^ chromatin.

## Results

### CENP-A^Cnp1^ Assembles on Noncentromeric DNA

The central domain of centromere 1 *(cen1)* is composed of the *cnt1* element, a portion of which is shared with *cen3,* and the *cen1*-specific *imr1* repeats that are virtually identical in sequence on the left and right sides [23]. The outer repeats flanking the central domain are also found at *cen2* and *cen3,* totaling 17–18 copies at centromeres [23,25].

The distribution of endogenous CENP-A^Cnp1^ across *cen1* was assessed using anti-CENP-A^Cnp1^ antiserum for chromatin immunoprecipitation (ChIP), confirming that endogenous CENP-A^Cnp1^ associates with the central domain *(cnt1* and *imr1),* but not with flanking outer repeats (Figure S1). To determine whether CENP-A^Cnp1^ chromatin forms on noncentromeric sequences in fission yeast, its association with a *ura4* ^+^ gene inserted at different sites in *cen1* was assessed using ChIP (Figure 1A). The use of *ura4^+^* insertions also provides specificity in ChIP for *cen1,* especially for duplicated *cnt1* and *imr1* regions and the more repetitive outer repeats. The *ura4-DS/E* allele (279-bp deletion) at its euchromatic locus provides an internal control for quantification. CENP-A^Cnp1^ shows no association with *ura4* ^+^ in the outer repeats of *cen1* (sites 1 and 2). No association of CENP-A^Cnp1^ is seen at a euchromatic control site *R.int-cnt1:ura4* ^+^ (R: between open reading frames SPBC342.01 and .02), even though in this case the *ura4^+^* gene is also flanked by 1.7 and 1.6 kb of DNA from the central domain of *cen1* (Figure S9)*.* However, just outside the tRNA^ala^ and tRNA^glu^ genes that demarcate the edge of the central domain, a 4-fold enrichment of CENP-A^Cnp1^ is detected (site 3). A similar level of CENP-A^Cnp1^ is detected inside the first tRNA^ala^ gene (site 4), but much higher levels (20- to 50-fold enrichment) of CENP-A^Cnp1^ are associated with *ura4* ^+^ in the core of the central domain of *cen1* (site 5 and 6). This confirms and extends previous analyses utilizing C-terminally 3 × HA tagged CENP-A [11] and is similar to the pattern of association seen for Mis6-HA across *cen1* [36].

These observations suggest that fission yeast CENP-A^Cnp1^ can associate with noncentromeric DNA inserted within the central domain. However, it is possible that the strong enrichment of *ura4* ^+^ in ChIPs is simply due to the association of CENP-A^Cnp1^ with adjacent centromeric DNA sequences in the IPs. To test this rigorously we used two strains in which 1.7 kb or 4.7 kb of DNA bearing the *ura4* ^+^ gene was inserted at site 6 in the middle of the central domain of *cen1, cnt1:ura4^+^*, and *cnt1:bigura4^+^*, respectively (Figure 1B). The sonicated chromatin used for anti-CENP-A^Cnp1^ ChIP was less than 800 bp (Figure 1C). The region within *ura4* ^+^ monitored by PCR is 0.9 kb and 0.5 kb (*cnt1:ura4* ^+^) or 2.2 kb and 2.2 kb (*cnt1:bigura4* ^+^) away from centromeric sequences, and thus, any enrichment of *ura4* ^+^ indicates association of CENP-A^Cnp1^ with *ura4* ^+^ DNA. Using this stringent assay, we observe that CENP-A^Cnp1^ assembles on *ura4* ^+^ DNA in *cnt1:ura4^+^* (19–35× enrichment) and even associates with the middle of *cnt1:bigura4* ^+^ which is at least 2 kb from any endogenous centromeric sequences (6–12× enrichment). Thus, we conclude that CENP-A^Cnp1^ can assemble on noncentromeric sequences.

The *R.int-cnt1:ura4* ^+^ control (R) consists of the *ura4* ^+^ gene flanked by 1.7 and 1.6 kb of central domain DNA [39], yet no association of CENP-A^Cnp1^ is seen at this location. Thus, central domain sequences alone are not sufficient to induce CENP-A^Cnp1^ assembly when placed on a chromosome arm. This is consistent with previous analyses showing that plasmids containing just central domain DNA do not exhibit centromere function or features associated with functional centromeres [23,24,27]. In addition to *ura4* ^+^, we have also detected CENP-A^Cnp1^ on *ade6* ^+^ when inserted in the central domain of centromeres (Figure S1). Thus, the deposition of CENP-A^Cnp1^ in Schizosaccharomyces pombe does not require specific underlying DNA sequences and can probably assemble on any noncentromeric DNA sequence provided that it is placed in the context of a functional centromere.

### Silencing Correlates with CENP-A^Cnp1^ Function and Levels in the Central Domain

The ChIP analyses above indicate that there is more CENP-A^Cnp1^ on *cnt1:ura4* ^+^ than on *cnt1:bigura4* ^+^. Both *ura4^+^* insertions at site 6 are silenced, as indicated by growth on counter-selective 5-fluoro-orotic-acid (FOA) plates. However, the *cnt1:ura4* ^+^ insertion exhibits more growth on FOA plates indicating that it is more strongly silenced than the larger *cnt1:bigura4* ^+^ insertion (Figure 1D). In support of this, more *ura4* ^+^ mRNA is detected in *cnt1:bigura4* ^+^ cells compared to *cnt1:ura4* ^+^ cells by reverse transcriptase (RT)-PCR (Figure 1E).

**Figure 2:**
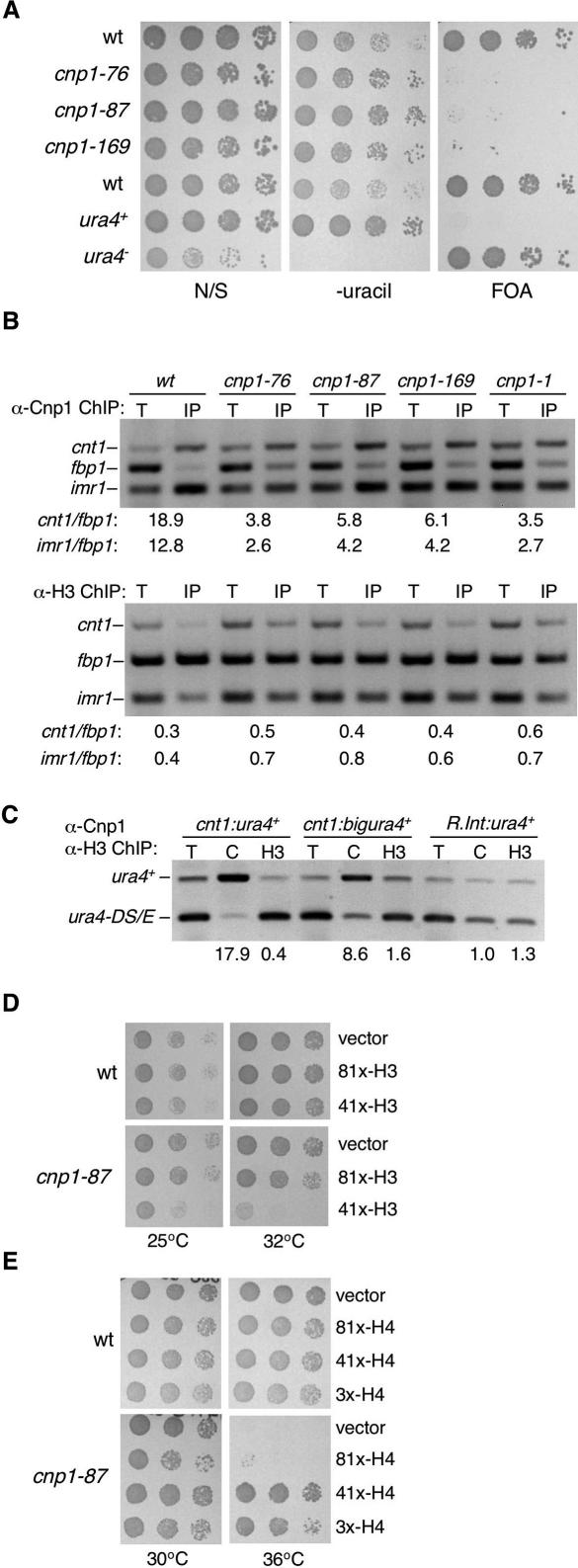
Silencing in the Central Domain Correlates with the Levels of Associated CENP-A^Cnp1^ and Histone H3 (A) Serial dilution of indicated strains. Strains contain *cnt1:ura4^+^* to assess central domain silencing. (B) ChIP analysis of CENP-A^Cnp1^ and H3 association with centromeric DNA in wild-type and indicated *cnp1* mutants using specific primers for *imr1, cnt1,* and *fbp1.* Enrichment of *imr1* and *cnt1* was assessed compared to *fbp1* in IP relative to total (T) extract. (C) ChIP analysis of CENP-A^Cnp1^ (C) and H3 (H3) association with *cnt1:ura4* ^+^ and *cnt1:bigura4* ^+^ insertions at site 6 and *R.int.cnt1:ura4* ^+^ (site R). (D) Serial dilution of wild-type and *cnp1–87* cells expressing additional histone H3 at low (prep81x-H3) or medium (prep41x-H3) levels, compared to empty vector, on minimal medium lacking leucine at 25 °C and 32 °C. (E) Serial dilution of wild-type and *cnp1–87* cells expressing additional histone H4 at low (prep81x-H4), medium (prep41x-H4), or high (prep3x-H4) levels, compared to empty vector, on minimal medium lacking leucine at 30 °C and 36 °C.

Consistent with the idea that silencing is due to spreading of CENP-A^Cnp1^ chromatin onto *ura4* ^+^ gene insertions, *cnp1* mutants alleviated silencing of *ura4* ^+^ within the central domain (less growth on FOA; Figure 2A) and less CENP-A^Cnp1^ was detectable by ChIP on the central domain (Figure 2B); this is supported by previous analyses [11,35,41]. The most severe *cnp1* alleles showed the greatest reductions in CENP-A^Cnp1^ levels: *cnp1–1*(L87Q) [11] *> cnp1–76* (T74M) *> cnp1–87* (E92K) *> cnp1–169* (V52A) (previously referred to as *sim2* mutants; [35]). This suggests that the production of defective CENP-A^Cnp1^ results in less CENP-A^Cnp1^ chromatin in the central domain, and that this chromatin state is more compatible with *ura4* ^+^ gene expression. ChIP with antibodies recognizing the C-terminus of histone H3 indicates that this is accompanied by increased incorporation of histone H3 into the central domain chromatin in all *cnp1* mutants (Figure 2B), and this concurs with analyses of other mutants affecting CENP-A^Cnp1^ deposition [41,42]. Silencing also correlates with chromatin composition in the centromere insertions: more H3 is associated with *cnt1:bigura4* ^+^ than with the more silent *cnt1:ura4^+^* (Figure 2C). Interestingly, in *Drosophila,* H3 can take the place of CENP-A^CID^ when CENP-A^CID^ levels are reduced [43].

In addition, *cnp1* mutants are sensitive to excess H3, displaying a correlation between allele severity and sensitivity to different H3 levels (Figures 2D and S2). Moreover, overexpression of histone H4 suppressed the temperature sensitivity of *cnp1* mutants, with higher levels of H4 being required to suppress more severe alleles (Figures 2E and S2), as reported for *cnp1–1* [42]. These genetic interactions suggest that additional H4 may assist in incorporation of mutant CENP-A^Cnp1^ into the central core by facilitating the formation of more H4/CENP-A^Cnp1^ heteromers. On the other hand, excess H3 would exacerbate the phenotype by competing with CENP-A^Cnp1^ for H4 from the available pool, further favoring H3/H4 deposition into central domain chromatin.

Together, these data imply that assembly of CENP-A^Cnp1^ chromatin on DNA inserted in the central domain is incompatible with gene expression and that when CENP-A^Cnp1^ is defective, histone H3 takes its place, allowing greater expression.

### Elevated CENP-A^Cnp1^ Levels Increase Central Domain Silencing and the Extent of CENP-A^Cnp1^ Chromatin

**Figure 3:**
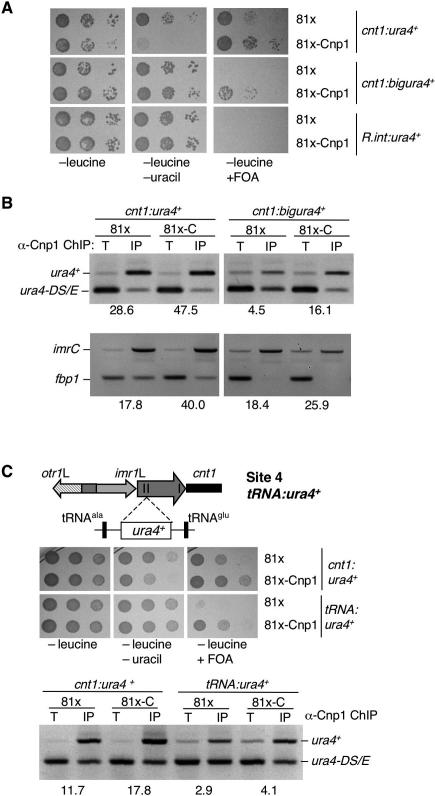
CENP-A^Cnp1^ Overexpression Enhances Silencing and Increases CENP-A^Cnp1^ Chromatin (A) Serial dilution of *cnt1:ura4^+^* or *cnt1:bigura4* ^+^ wild-type cells overexpressing CENP-A^Cnp1^ (prep81x-Cnp1) compared with empty vector (prep81x) on indicated media. (B) ChIP analysis of CENP-A^Cnp1^ association with *cnt1:ura4* ^+^ and *cnt1:bigura4* ^+^ (top) or centromeric DNA (*imrC,* bottom) in wild-type cells overexpressing CENP-A^Cnp1^ or nothing from prep81x. Enrichment of *ura4* ^+^ and *imrC* was assessed compared to *ura4-DS/E* and *fbp1,* respectively, in IP relative to total (T) extract*.* (C) Serial dilution of wild-type cells containing *cnt1:ura4^+^* or *imr1LtRNA:ura4* ^+^ (inserted at site 4 between tRNA^ala^ and tRNA^glu^ as shown) overexpressing CENP-A^Cnp1^ or nothing from prep81x on indicated media (top). ChIP analyses of CENP-A^Cnp1^ association with *cnt1:ura4* ^+^ (site 6) and *imr1LtRNA:ura4* ^+^ (site 4) insertions in wild-type cells overexpressing CENP-A^Cnp1^ or nothing from prep81x (bottom).

Since defective CENP-A^Cnp1^ alleviates silencing, we determined whether, conversely, overexpression of CENP-A^Cnp1^ causes an increase in central domain silencing. Unlike budding yeast, fission yeast are able to tolerate overexpression of CENP-A^Cnp1^, to an extent that the normally undetectable CENP-A^Cnp1^ is detectable by western analysis [44] (Figure S3). Overexpression of CENP-A^Cnp1^ (from the attenuated *nmt1* promoter in prep81x) in a wild-type strain bearing *cnt1:ura4* ^+^ or *cnt1:bigura4* ^+^ increased the level of silencing, as indicated by increased growth on FOA (Figure 3A), and more CENP-A^Cnp1^ was detected on *cnt1:ura4^+^* and *cnt1:bigura4* ^+^ (Figure 3B). This suggests that the *ura4^+^* sequence inserted in the central domain is not saturated for CENP-A^Cnp1^. There was also a detectable increase in the levels of CENP-A^Cnp1^ on endogenous central domain sequences (Figure 3B).

Most CENP-A^Cnp1^ is normally confined to the central domain, which is flanked on both sides by tRNA^ala^ and tRNA^glu^ genes, separated by 349 bp [37]. When CENP-A^Cnp1^ was overexpressed, increased silencing (more growth on FOA) occurred at site 4 (*ura4^+^* between the tRNA^ala^ and tRNA^glu^ genes; Figure 3C), and a subtle increase in CENP-A^Cnp1^ levels was reproducibly detected on *ura4^+^* at this site. However, in strains overexpressing CENP-A^Cnp1^, additional CENP-A^Cnp1^ could not be detected in the heterochromatin domain beyond the tRNA^ala^ and tRNA^glu^ genes (unpublished data). Moreover, there was no apparent effect on the extent of the CENP-A^Cnp1^ domain in a strain lacking heterochromatin on the outer repeats (*clr4Δ;* unpublished data). These data suggest that CENP-A^Cnp1^ chromatin is confined to the central domain but that overexpression of CENP-A^Cnp1^ allows some expansion in the extent of the CENP-A^Cnp1^ domain beyond the proximal tRNA^ala^ gene into an inserted *ura4^+^* gene.

### Overexpression of Histone H3 Results in Defective CENP-A^Cnp1^ Chromatin

**Figure 4:**
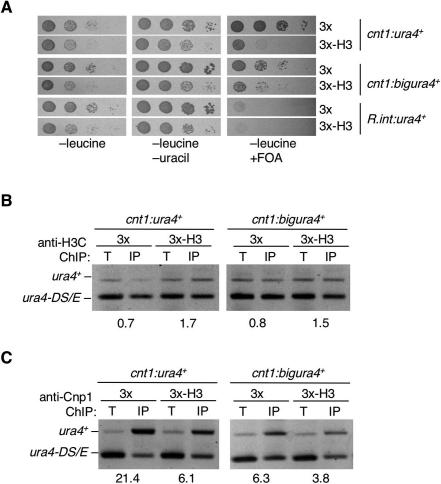
Histone H3 Overexpression Alleviates Silencing and Decreases CENP-A^Cnp1^ Levels in the Central Domain (A) Serial dilution of *cnt1:ura4^+^* or *cnt1:bigura4* ^+^ wild-type cells overexpressing histone H3 or nothing from prep3x on indicated media. (B) ChIP analysis of histone H3 association with *cnt1:ura4* ^+^ and *cnt1:bigura4* ^+^ in wild-type cells overexpressing H3 or nothing from prep3x. (C) ChIP analysis of CENP-A^Cnp1^ association with *cnt1:ura4* ^+^ and *cnt1:bigura4* ^+^ in wild-type cells overexpressing H3 or nothing from prep3x.

As the degree of central domain silencing correlates with increased H3 levels in both *cnp1* mutants and *cnt1:bigura4^+^* versus *cnt1:ura4* ^+^ *,* we investigated the effects of simply overexpressing H3 on central domain silencing and composition. Additional histone H3 was expressed from the *nmt1* promoter (prep3X-H3) in strains harboring *cnt1:ura4^+^* or *cnt1:bigura4* ^+^ (Figure 4A). Quantitative western analyses indicate that 2- to 3-fold more H3 is present and that the levels of CENP-A^Cnp1^ are unaffected (Figures S4 and S5). H3 overexpression resulted in increased *ura4* ^+^ expression from *cnt1:ura4* ^+^ as shown by loss of growth on FOA. This was accompanied by increased H3 on *cnt1:ura4* ^+^ and *cnt1:bigura4* ^+^ (Figure 4B) and a concomitant decrease in CENP-A^Cnp1^ levels (Figure 4C).

### Alteration of the H3:H4:CENP-A^Cnp1^ Ratio Perturbs the Integrity of Central Domain CENP-A^Cnp1^ Chromatin

**Figure 5:**
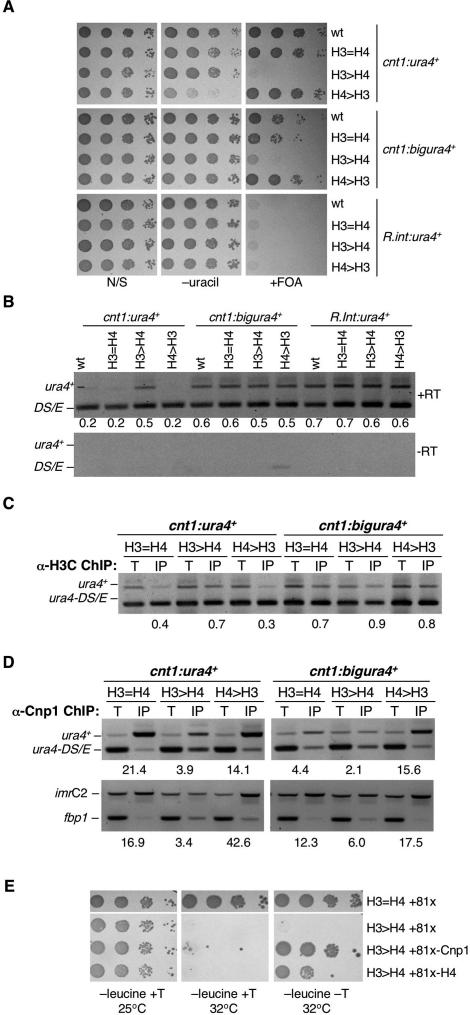
Altered H3:H4 Gene Ratio Affects Silencing and CENP-A^Cnp1^ Deposition in the Central Domain (A) Serial dilution of wild-type cells (H3:H4 3:3), H3 = H4 (H3:H4 2:2), H3 > H4 (H3:H4 2:1), H4 > H3 (H3:H4 1:2) containing *cnt1:ura4^+^* or *cnt1:bigura4* ^+^ on indicated media. (B) RT-PCR to assess levels of *ura4* expression relative to *ura4-DS/E* in H3 = H4 (H3:H4 2:2), H3 > H4 (H3:H4 2:1), and H4 > H3 (H3:H4 1:2) cells containing *cnt1:ura4^+^* or *cnt1:bigura4* ^+^. Relative levels of *ura4^+^* mRNA are indicated. (C) ChIP analysis of histone H3 association with *cnt1:ura4* ^+^ and *cnt1:bigura4* ^+^ in H3 = H4, H3 > H4, and H4 > H3 cells. (D) ChIP analysis of CENP-A^Cnp1^ association with *cnt1:ura4* ^+^ and *cnt1:bigura4* ^+^ (top) and centromeric DNA (*imr1C,* bottom) in H3 = H4, H3 > H4, and H4 > H3 cells. (E) Temperature-sensitive growth of H3 > H4 at 32 °C is suppressed by expression of additional CENP-A^Cnp1^ or histone H4. Serial dilution growth assay of wild-type H3 = H4 (H3:H4 2:2) or H3 > H4 (H3:H4 2:1) cells overexpressing CENP-A^Cnp1^, H4, or nothing from prep81x on −leucine +Thiamine (*nmt1* promoter repressed), −leucine −Thiamine (expressed) at 25 °C or 32 °C as indicated.

The *nmt1* promoter used in the above experiments is active throughout the cell cycle whereas histone gene expression is normally induced from endogenous promoters in early S phase [11,45]. To rule out the possibility that the observed effects were due to constitutive expression of H3 from the *nmt1* promoter, we altered the ratio of H3:H4 genes while retaining the normal endogenous promoter elements. In fission yeast, three pairs of H3 and H4 genes are transcribed divergently from a putative common regulator at three distinct loci (H3.1/H4.1, H3.2/H4.2, and H3.3/H4.3) [46]. We completely deleted the H3.1/H4.1 pair and either the H3.2 gene, resulting in a H3:H4 gene ratio of 1:2 (H4 > H3), or the H4.2 gene, resulting in a H3:H4 gene ratio of 2:1 (H3 > H4) [47] (Figure S6). Increased H3 relative to H4 (H3 > H4) alleviated silencing of both *cnt1:ura4^+^* and *cnt1:bigura4^+^*, resulting in failure to grow on counter-selective FOA plates (Figure 5A), indicating a complete inability to silence *ura4^+^* expression. Conversely, excess H4 (H4 > H3) enhanced silencing of *cnt1:ura4* ^+^ as indicated by reduced growth on plates lacking uracil. These phenotypic effects were confirmed by RT-PCR analysis (Figure 5B); more *ura4* mRNA is produced from *cnt1:ura4* ^+^ in the H3 > H4 strain (although we were unable to detect a further increase in the comparatively high levels of *ura4* mRNA in cells containing *cnt1:bigura4^+^*). These analyses also indicate that central domain silencing is fully intact in the control strain (H3 = H4; H3:H4 ratio 2:2) used in these experiments (Figure 5A and 5B; compare to wild type; H3:H4 ratio 3:3). The strain also displays robust ChIP of CENP-A^Cnp1^ on endogenous centromeric sequences and *ura4^+^* sequences inserted therein (e.g., Figure 5D). Thus (as with *cnp1* mutants), disturbing the H3:H4 balance so that more H3 is expressed alleviates silencing in the central domain, while additional H4 enhances silencing.

To determine the impact of additional H3 on centromere integrity, we assessed CENP-A^Cnp1^ association with centromeres by ChIP and immunolocalization. Cells with a histone imbalance in favor of H3 (H3 > H4) were confirmed to have increased H3 levels on *cnt1:ura4^+^* and *cnt1:bigura4* ^+^ by ChIP (Figure 5C). CENP-A^Cnp1^ levels were greatly reduced on *cnt1:ura4^+^* and *cnt1:bigura4* ^+^ in cells with excess H3 (Figure 5D), yet the cellular levels of CENP-A^Cnp1^ are unaffected (Figure S5). Lower levels of CENP-A^Cnp1^ are normally detected on the *ura4^+^* region of *cnt1:bigura4* ^+^ relative to *cnt1:ura4* ^+^, and excess histone H3 caused a further decrease in the level of CENP-A^Cnp1^ associated with the middle of the *cnt1:ura4* ^+^ and a consistent but less dramatic effect on *cnt1:bigura4^+^* (Figure 5D). We also observed a reduction of CENP-A^Cnp1^ levels on endogenous centromeric sequences in strains with excess H3 (Figure 5D). In strains with an excess of H4 relative to H3 (H4 > H3), more CENP-A^Cnp1^ was detected on *cnt1:bigura4* ^+^, although the effects on endogenous centromeric sequences and *cnt1:ura4^+^* were more variable (Figure 5D and unpublished data).

**Figure 6:**
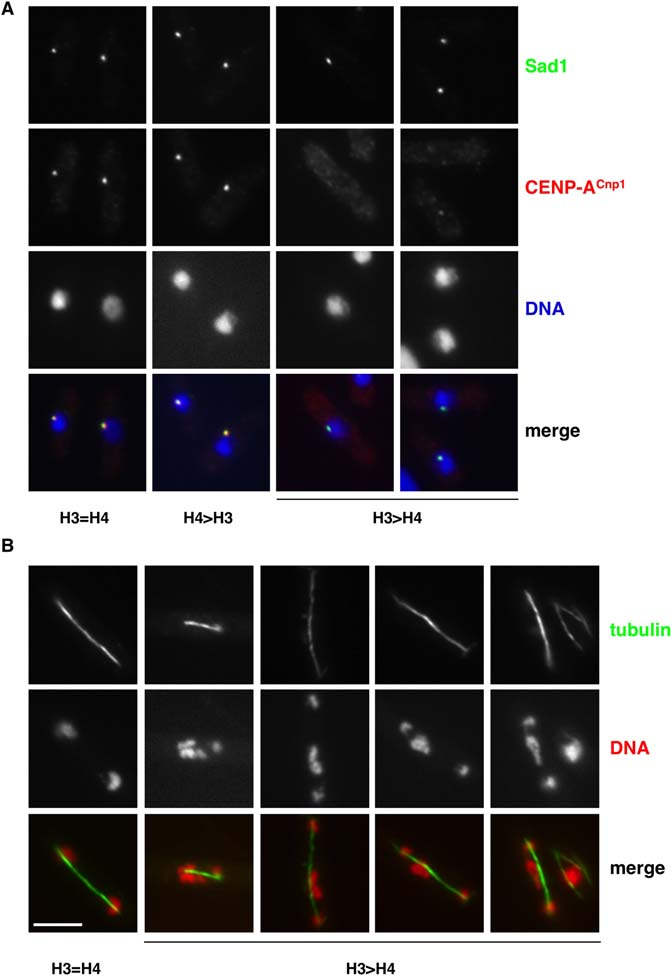
Excess H3 Causes Delocalization of CENP-A^Cnp1^ and Chromosome Missegregation (A) H3 = H4, H3 > H4, and H4 > H3 cells stained with anti-Sad1 to decorate the SPB (red), anti-CENP-A^Cnp1^ (green), and DAPI (DNA: blue). (B) H3 = H4 and H3 > H4 cells stained with anti-α-tubulin to decorate mitotic spindles (green) and DAPI (DNA: red). Scale bar, 5 μm.

Chromatin immunoprecipitation of a protein reports the population average for its association with particular DNA sequences. To assess CENP-A^Cnp1^ levels in individual cells, immunolocalization was performed. In fission yeast, the three centromeres cluster adjacent to the spindle pole body (SPB) in interphase and this was used as a marker for centromere position. In both H3 = H4 and H4 > H3 strains a clear CENP-A^Cnp1^ signal (red) was detected next to the SPB (green) in all cells (Figure 6A). However, in H3 > H4 cells, the CENP-A^Cnp1^ signal was weaker or undetectable and displayed variation between cells. The majority of cells had much weaker staining than H3 = H4 (54% versus 7%), and very few displayed bright/very bright staining (9% versus 93% in H3 = H4). In addition, the CENP-A^Cnp1^ signal in H4 > H3 cells was quantified and found to be of greater intensity than in H3 = H4 cells (2.5-fold brighter on average in the H4 > H3 cells compared to the H3 = H4 cells; details of quantification methods described in Figure S7 and Materials and Methods). Thus, the immunolocalization data confirm the ChIP analyses and indicate that the level of CENP-A^Cnp1^ at centromeres in H3 > H4 cells exhibits variation within the population.

These observations are consistent with a scenario in which excess CENP-A^Cnp1^ allows more CENP-A^Cnp1^/H4 chromatin assembly in the central domain, while elevated H3 levels permit more H3/H4 nucleosomes to occupy the central domain at the expense of CENP-A^Cnp1^. It also appears that an increase in the available H4 pool allows CENP-A^Cnp1^ to compete more effectively with H3 for incorporation in the centromere.

### Proper Chromosome Segregation Is Dependent on Maintenance of Histone Ratios

Altering the ratios of H3:H4:CENP-A^Cnp1^ have clear effects on central domain chromatin, but what are the consequences for chromosome segregation? It is possible that a functional kinetochore can be assembled despite alterations in the proportion of CENP-A^Cnp1^/H3 in the central domain. To determine whether chromosome segregation is aberrant in strains with altered histone ratios, fixed cells were stained for chromosomal DNA (DAPI: red) and anti-α-tubulin to identify cells with a mitotic spindle (green) (Figure 6B). The H3 > H4 strain exhibited significantly higher rates of chromosome segregation defects in anaphase (lagging chromosomes and uneven segregation) compared to the H4 > H3 and control (H3 = H4) strains (16.6%, 0%, and 1.4%, respectively, see Figure S8). Together these data suggest that excess H3 interferes with the localization of CENP-A^Cnp1^ at centromeres resulting in defective kinetochore function during mitosis.

It could be argued that disturbing histone ratios in the cell is likely to have pleiotropic effects that lead to chromosome missegregation. For instance, mutants that affect silencing and function of centromeric outer repeat chromatin display high frequencies of lagging chromosomes. However, alleviation of outer repeat silencing was not observed when H3 was overexpressed or in H3 > H4 strains (unpublished data). In addition, prep81x-Cnp1 rescued defective growth of the H3 > H4 strain, allowing growth at 32 °C (Figure 5E), suggesting that the primary defect in H3 > H4 cells is in central domain function. As expected, the defective growth of the H3 > H4 strain was also rescued by H4 overexpression (Figure 5E).

### Other Kinetochore Proteins Can Associate with Noncentromeric DNA and Are Affected by Histone Ratio

**Figure 7:**
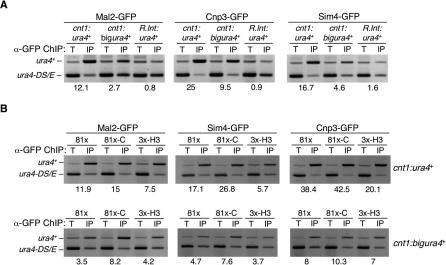
Kinetochore Proteins Associate with Noncentromeric DNA and Their Association Is Altered by Overexpression of CENP-A^Cnp1^ or H3 (A) ChIP analysis of Mal2-GFP, CENP-C^Cnp3^-GFP, or Sim4-GFP association with *cnt1:ura4* ^+^, *cnt1:bigura4* ^+^ insertions at site 6 or *R.int.cnt1:ura4* ^+^ (site R). Enrichment of *ura4* ^+^ was assessed compared to *ura4-DS/E* in IP relative to total (T) extract*.* (B) ChIP analysis of Mal2-GFP, CENP-C^Cnp3^-GFP, or Sim4-GFP association with *cnt1:ura4* ^+^, *cnt1:bigura4* ^+^ in wild-type cells overexpressing CENP-A^Cnp1^ (prep81x-C), H3 (prep3x-H3), or nothing (prep81x).

Mutations in *cnp1* or genes encoding kinetochore proteins alleviate silencing in the central domain [31,35,36]. Previous analyses suggest that the kinetochore proteins Mis6 and Sim4 can associate with *cnt1:ura4^+^* [35,36]. To examine this further, ChIP was used to determine if three kinetochore proteins, CENP-C^Cnp3^-GFP, Mal2-GFP, and Sim4-GFP, are also associated with the middle of *cnt1:ura4^+^* and *cnt1:bigura4* ^+^. Under conditions where chromatin was extensively sheared, all three kinetochore proteins were found to associate with the middle of the *ura4* ^+^ gene when it was inserted within *cnt1* at site 6 (both *cnt1:ura4^+^* and to a lesser degree for *cnt1:bigura4* ^+^), but not when it was flanked by 1.7 and 1.6 kb of central domain DNA at a euchromatic site (R: *R.int-cnt1:ura4^+^*) (Figure 7A).

Thus, not only CENP-A^Cnp1^, but three other kinetochore proteins tested associate with noncentromeric DNA when inserted in the context of a functional fission yeast centromere.

Elevated levels of CENP-A^Cnp1^ or histone H3 have opposite effects, respectively leading to more and less deposition of CENP-A^Cnp1^ on *cnt1:ura4* ^+^ and *cnt1:bigura4* ^+^. Since CENP-A^Cnp1^ is required for kinetochore assembly, we tested whether the levels of Mal2-GFP, Sim4-GFP, and CENP-C^Cnp3^-GFP on *cnt1:ura4* ^+^ and *cnt1:bigura4* ^+^ are also affected in strains expressing additional CENP-A^Cnp1^ or histone H3 (Figure 7B). Cells expressing additional CENP-A^Cnp1^ reproducibly showed more CENP-C^Cnp3^-GFP, Mal2-GFP, and Sim4-GFP associated with *ura4^+^* in the central core, especially for *cnt1:bigura4* ^+^. Conversely, in cells expressing more histone H3, less of these three kinetochore proteins were detected on *cnt1:ura4* ^+^. This indicates that adjusting the levels of CENP-A^Cnp1^ and H3 not only alters the extent and density of CENP-A^Cnp1^ chromatin at centromeres but also influences the recruitment of other kinetochore proteins, resulting in defective chromosome segregation.

## Discussion

We have found that CENP-A^Cnp1^ in fission yeast can associate with noncentromeric sequences provided that they are placed in an environment with the required contextual cues for CENP-A^Cnp1^ chromatin assembly. Our analyses show that excess CENP-A^Cnp1^ (or H4) allows the deposition of more CENP-A^Cnp1^ at the expense of H3 while an excess of H3 allows the deposition of more H3 in place of CENP-A^Cnp1^ and leads to defective chromosome segregation. These analyses strongly support the concept that deposition of CENP-A^Cnp1^ in the central kinetochore domain exhibits surprising plasticity in that it can be disturbed or enforced simply by changes in the ratios of H3, H4, and CENP-A^Cnp1^. These observations indicate that maintenance of the unique chromatin composition of the central domain is vital in ensuring proper kinetochore assembly and function. Thus, both the functional state of CENP-A^Cnp1^ and its density are important for centromere function.

In metazoa, the assembly of CENP-A chromatin and kinetochores is plastic [1,2,13]. In humans, CENP-A and kinetochores are normally associated with a subset of the centromeric alpha-satellite repeats. However, CENP-A can assemble at locations on chromosome arms lacking alpha-satellite DNA, resulting in the formation of neocentromeres [14]. In *Drosophila,* experiments with truncated minichromosomes derived from the X chromosome demonstrate that CENP-A^CID^ and kinetochore proteins can spread into chromosomal regions where they do not normally associate and then act as a functional neocentromere when the X centromere is subsequently deleted [20]. In addition, overexpression of CENP-A^CID^ in *Drosophila* cells can attract other kinetochore proteins and even direct microtubule association at noncentromeric locations [22].

The similarity in the organization of centromeric DNA at all fission yeast centromeres suggested that, like *Saccharomyces cerevisiae,* the assembly of CENP-A^Cnp1^ chromatin might be more DNA sequence–dependent and thus less pliable than in metazoa [23,25]. The identification of Ams2, a GATA-like DNA-binding factor that affects CENP-A^Cnp1^ deposition, supported this possibility [42]. However, here we have shown that, as observed in *Drosophila* and human cells, fission yeast CENP-A^Cnp1^ can spread into and associate with additional sequences inserted within the central domain. Thus, provided the right contextual cues exist, fission yeast CENP-A^Cnp1^ chromatin can potentially associate with any DNA sequence. The establishment and continued loading of CENP-A in the central domain may be dependent on the central domain sequences themselves, but once it has been initiated, our analyses indicate that it can engulf inserted noncentromeric DNA. In addition, the association of CENP-A^Cnp1^ with exogenous sequences in the central domain is accompanied by the recruitment of other known kinetochore proteins. It is also possible that the association of CENP-A with *ura4* DNA in the central domain might reflect some higher order structure within the centromere.

In *S. cerevisiae,* additional CENP-A^Cse4^ is degraded and only nondegradable mutant protein can be overexpressed [44]. In fission yeast there appears to be no inherent difficulty in overexpressing CENP-A^Cnp1^ since increased CENP-A^Cnp1^ is detected by western analysis upon overexpression (Figure S3); and this is the cause of increased CENP-A^Cnp1^ incorporation into the central domain. Conversely, elevated levels of H3 lead to more H3 and less CENP-A^Cnp1^ in central domain chromatin. In addition, mutations in CENP-A^Cnp1^ are antagonized by H3 but rescued by H4 overexpression, and defects exhibited by H3 > H4 cells are suppressed by additional CENP-A^Cnp1^. This indicates that perturbation in the relative levels of CENP-A^Cnp1^, H3, and H4 can adversely affect the composition of CENP-A^Cnp1^ chromatin assembled at centromeres, affecting the relative density of CENP-A^Cnp1^/H4 to H3/H4 nucleosomes. Disturbing the normal CENP-A:H3:H4 ratio clearly results in defective kinetochore function and chromosome segregation. Consistent with this, decreased CENP-A^Cnp1^ at centromeres in cells lacking Ams2 is suppressed or antagonized by overexpression of H4 and H3, respectively [41,42].

**Figure 8:**
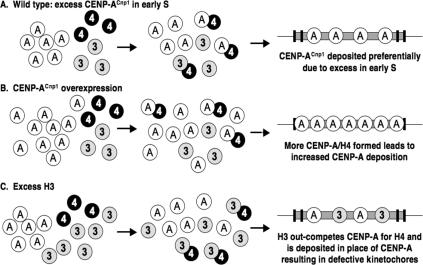
Model: Influence of CENP-A^ Cnp1^ and Histone H3 Levels on CENP-A^Cnp1^ Deposition Normally CENP-A^Cnp1^ expression peaks prior to H3/H4 expression in early S phase allowing it to compete with H3 for the limited H4 pool to form CENP-A^Cnp1^/H4 heteromers. Overexpression of CENP-A^Cnp1^ allows it to compete more efficiently with H3 for H4, leading to the deposition of more CENP-A^Cnp1^ in the central domain. Excess H3 reduces the H4 pool available to form CENP-A^Cnp1^/H4 heteromers, and consequently, H3 rather than CENP-A^Cnp1^ is deposited in the central domain. This leads to loss of kinetochore integrity and chromosome missegregation.

In fission yeast, it is likely that H3 and H4 mRNAs are coordinately expressed early in S phase from a common regulatory element residing between their divergent promoters, whereas CENP-A^Cnp1^ mRNA is expressed from late M, peaking prior to S phase [11,41,45,46]. This suggests that normally CENP-A^Cnp1^ is available before maximal H3 and H4 expression. As fission yeast centromeres are replicated early in S phase [48,49], this difference in expression timing may enhance the effective concentration of CENP-A^Cnp1^ relative to H3 at the start of S phase, and thus allow it to compete more effectively with the initially low levels of newly produced H3 for assembly with new H4 (Figure 8). Our data suggest that altering the ratio of H3:H4 either by altering the normal gene number from equivalency (3:3 or 2:2) to 2:1, or overexpressing H3, provides more H3 to titrate out the pool of H4 available for assembly with CENP-A^Cnp1^ into chromatin. Conversely, excess H4 relative to H3 (H3:H4 1:2) increases the pool of H4 available for assembly into chromatin with CENP-A^Cnp1^, leading to more CENP-A^Cnp1^ at centromeres. These data suggest that the relative amounts of H3, H4, and CENP-A are normally delicately balanced in the cell to allow normal CENP-A^Cnp1^ chromatin and kinetochore assembly. Changes in this balance, the timing, or amount of expression can lead to increased or decreased CENP-A^Cnp1^ chromatin at centromeres (Figure 8). Precise regulation of histone levels, e.g., coregulation of H3 and H4, is likely to be important in other organisms (including human) where CENP-A overexpression has been detected in the tissue from colorectal tumors [50]. In *S. cerevisiae,* elevated levels of histone H3 and H4 cause defective chromosome segregation [51]; it is possible that this is due to defects in deposition of CENP-A^Cse4^ at centromeres.

The extent of sequence occupied by CENP-A^Cnp1^ chromatin appears to be flexible and increases in response to higher levels of CENP-A^Cnp1^. The tRNA genes flanking the central domain act as a barrier preventing heterochromatin seeping into the kinetochore domain [37]. Here, we detect more CENP-A^Cnp1^ on *ura4^+^* between the tRNA^ala^ and tRNA^glu^ when CENP-A^Cnp1^ is overexpressed but not distal to tRNA^ala^ in the heterochromatin domain (Figure 3C); thus, CENP-A^Cnp1^ chromatin is still mainly confined to the central domain.

The mechanism of CENP-A assembly into centromeric chromatin is not known. In mammalian cells, CENP-A is synthesized in G2 and thereby separated from bulk histone synthesis [52,53]. Such analyses suggested that CENP-A chromatin assembly is uncoupled from replication. One model suggests that the CENP-A nucleosomes are randomly segregated at S phase and are subsequently recognized by the CENP-A chromatin assembly machinery resulting in the deposition of neighboring new CENP-A during interphase [2,52,53]. In fission yeast, in addition to a replication-independent pathway, there appears to be a replication-coupled pathway that allows CENP-A^Cnp1^ chromatin assembly [11,41]. Deposition during S phase is coupled with the expression of new histones (dependent on Ams2) while G2 assembly requires Mis6 [11,41,42]. Higher levels of H3 interfere with CENP-A^Cnp1^ incorporation at centromeres, suggesting that excess H3 can overwhelm the normal assembly pathways to predominate in the central domain. Surprisingly, there is no inherent impediment to deposition of H3 in the central domain. If a mechanism exists to prevent the over-incorporation of H3 in the central domain, it is easily overwhelmed. It is possible that H3 is incorporated during S phase but can be subsequently replaced by CENP-A^Cnp1^ via a replication-independent mechanism operating throughout the cell cycle. We envisage a scenario in which excess H3 results in overloading of the central domain with H3 nucleosomes, which in turn interferes with the recognition of central domain chromatin by activities that evict H3 and replace it with CENP-A^Cnp1^. Thus, the balance between H3, H4, and CENP-A^Cnp1^ levels is critical to the incorporation of new CENP-A^Cnp1^ nucleosome and kinetochore function.

## Materials and Methods

### Yeast strains.

S. pombe strains are listed in Table S1. Standard procedures were used for growth and genetic manipulations [54]. To construct FY4638 (*cnt1:bigura4^+^*), a 4.7-kb fragment of DNA containing the *ura4^+^* gene (1.7 kb) flanked by 1.3 kb and 1.7 kb of DNA from the *ade6^+^* locus on the left and right side, respectively [36], was introduced at *cnt1* by transforming strain FY319 (*cnt1:ade6^+^*) [39]. Correct integrants *(cnt1:ade6-ura4^+^-ade6)* were identified by growth on plates lacking uracil and red color on limiting adenine and confirmed by PCR.

Crosses were performed using existing strains containing altered numbers of histone H3/H4 genes: FY3569 (H3:H4, 2:2), FY4753/4754 (H3:H4, 2:3), and FY4755/4756 (H3:H4, 3:2) [47] (see Table S1) to obtain the imbalanced strains FY7488 (H3:H4, 1:2) and FY7450 (H3:H4, 2:1). The imbalanced strains FY4813 (H3:H4, 2:3) and FY4816 (H3:H4, 3:2) were obtained by replacing the *ura4^+^* gene from FY4753 (H3:H4, 2:3) and FY4755 (H3:H4, 3:2) by a 2.2-kb *LEU2^Sc^* fragment amplified using M32 and M35 primers (see Table S2). To obtain the imbalanced strains FY7370 (H3:H4, 1:2) and FY7372 (H3:H4, 2:1), FY3569 was crossed to FY4813 or FY4816, respectively.

*cnp3^+^* was C-terminally tagged in the genome with green fluorescent protein using a PCR-based method [55].

### Plasmids and primers.

Primers used in this study are listed in Table S2.

Histone H3.2, histone H4.2, and *cnp1* ^+^ open reading frames were amplified using the following primers: H3.2-XhoI-RI, H3.2–3-Xho-Bam, H4.2–5-Xho-Bam, H4.2–3-Xho-Bam, cnp1–5-XhoI, and cnp1–3-Xho-Bam and inserted into BamHI/XhoI digested pREP plasmids. For overexpression studies, pRep81X-Cnp1, pRep3X-H3, and pRep3X-H4 were used.

### RT-PCR.

Total RNA was prepared from strains grown in YES at 25 °C and RT-PCR performed as described [56]. Primers WA41 and WA42 (Table S2) were used for amplification and quantitation performed as described [35].

### ChIP.

ChIP was performed as described [57] except for the following modifications: For Cnp1 and H3C ChIPs, cells were fixed 1% PFA for 20 min at room temperature. For GFP ChIPs, cells were grown at 25 °C, incubated 2 h at 18 °C, and then fixed for 30 min at 18 °C with 3% PFA. Cells were spheroplasted at 1 × 10^8^ cells/ml in PEMS + 0.4 mg/ml zymolyase-100T (MP Biomedicals, http://www.mpbio.com) for 40 min at 36 °C. Cells were washed twice in PEMS and cell pellets frozen at 80 °C. The chromatin was sheared using either a MSE Soniprep 150 (SANYO, http://sanyo.com) sonicator (3 times 17 s, maximum amplitude) or the Bioruptor (Diagenode, http://www.diagenode.com) sonicator (20 min, 30 s ON and 30 s OFF at “High” [200 W] position). The extent of the shearing was checked either by ethidium bromide on a 1.7% agarose gel or by Southern blot (using *ura4^+^* probe). The sonicated chromatin used for ChIP was less than 800 bp. 10 μl of α-Cnp1 antiserum [58], 2–4 μl of α-H3C antibody (Abcam, ab1791; http://www.abcam.com), and 1.5 μl of α-GFP antibody (Invitrogen, A-11122; http://www.invitrogen.com) were used in ChIPs. Multiplex PCR analysis was performed as described [31]. PCR products were quantified as described for RT-PCR. For the input PCR, the *cnt, imr,* and *otr* values were normalized to the *fbp* value, giving the “input ratio.” Enrichment of *cnt* (WA26-WA27 primers pair, F7cnt1-R9cnt1 primers pair, and F10cnt1-R12cnt1 primers pair, Table S2), *imr* (WA28-WA29 primers pair, imrEf-imrEr primers pair, or imrC2f-imrC2r primers pair, Table S2) and *otr* (WA31-WA32 primers pair and CenF-CenR primers pair, Table S2) bands in the ChIPs was calculated relative to the *fbp* (WA33-WA34 primers pair, Table S2) band and then corrected for the ratio obtained in the input PCR. ChIPs performed on strains with *ura4* ^+^ insertions at centromere 1 were analyzed by PCR as described [56]. ChIP experiments were performed two to five times; representative examples are presented.

### Cytology.

Immunolocalization was performed as described [59]. Cells were grown at 32 °C and fixed for 5–10 min in 3.7% freshly prepared formaldehyde for staining with α-Cnp1 and α-Sad1 (provided by I. Hagan) antibodies or fixed for 10–15 min in 3.7% formaldehyde, 0.05% glutaraldehyde for immunolabeling of microtubules. The following antibodies were used: sheep α-Cnp1 antiserum (1:300), rabbit α-Sad1 (1:50), mouse TAT1 α-tubulin tissue culture supernatant (from I. Hagan and K. Gull) (1:15). Alexa Fluor 594 (Invitrogen, A11016) or Alexa Fluor 488 (Invitrogen, A11029 or A21441) conjugated secondary antibodies were used at 1:1,000. Microscopy was performed as described [59] using a Zeiss Imaging 2 microscope (Zeiss, http://www.zeiss.com). Image acquisition was controlled using Metamorph software (Universal Imaging Corporation, http://www.moleculardevices.com).

### Quantification of Cnp1 staining.

Comparison of Cnp1 signal intensity in H3 = H4 (2:2) versus H4 > H3 was performed using the following procedure: Sad1 staining served as an internal control for staining efficiency and a marker for the approximate position of the centromeres (which cluster adjacent to the SPB). Image capture and analysis was performed using Metamorph software. Random fields of cells were captured using identical exposures for all samples (2 s for Sad1 and 0.5 s for Cnp1). Only G2 cells in which the Sad1 (SPB) signal was in focus were included in quantification of Sad1/Cnp1 spots. A circular region of interest (ROI) of 0.77 μm diameter was drawn around these Sad1 spots. In addition, ten ROIs per field were randomly placed on cells to measure background signal. All ROIs were then transferred to the Cnp1 image. If necessary, the position of ROIs was altered slightly (Sad1 and Cnp1 signals are adjacent or overlapping). Total signal intensity was measured for each ROI. Mean background intensity was calculated for each channel and subtracted from the mean of Sad1 or Cnp1 spot intensities as appropriate. The mean corrected Cnp1 intensity was divided by the mean corrected Sad1 intensity to normalize for efficiency of staining, giving a final value for Cnp1 staining intensity. To determine the relative Cnp1 staining intensity, the value for the H4 > H3 strain was divided by the value for the H3 = H4 strain. This method was, however, determined not to be appropriate for measurement of relative Cnp1 signal intensity in H3 > H4 cells. This was because in many cells it was not possible to place the Cnp1 ROI with confidence due to very low intensity or undetectable Cnp1 staining at centromeres. Therefore, the Cnp1 signals were simply categorized instead. Images were obtained as above, and then larger ROI circles of 1.16 μm diameter were drawn around Sad1 spots that fit the criteria described above. The ROIs were then transferred to the Cnp1 images. If a Cnp1 spot could be identified within this ROI, it was classified as “very bright,” “bright,” or “faint” according to the maximum intensity within the spot. If no spot could be distinguished above background, it was recorded as “undetectable.”

## Supporting Information

Figure S1 CENP-A^Cnp1^ Chromatin Is Confined to the Central Domain and Can Associate with Noncentromeric DNA (247 KB DOC)

Figure S2 Correlation between Strength of *cnp1* Alleles (Severity of Temperature-Sensitive Phenotype) and Sensitivity to or Suppression by Different Levels of H3 and H4, Respectively (338 KB DOC)

Figure S3 CENP-A^Cnp1^ and Histone H3 Can Be Detected When Overexpressed from prep3x and Anti-H3-Cterm Does Not Cross-React with CENP-A^Cnp1^
(1.4 MB DOC)

Figure S4 Total Histone H3 and H4 Levels Are Elevated 2.5- and 6-Fold When Overexpressed (191 KB DOC)

Figure S5 Levels of 13× Myc-CENP-A^Cnp1^ Are Unaffected by Expression of Histone H3 or H4 (378 KB DOC)

Figure S6 Construction of Strains with Altered H3:H4 Gene Ratios (216 KB DOC)

Figure S7 Excess H3 Causes Delocalization of CENP-A^Cnp1^: Quantification of CENP-A^Cnp1^ Staining (49 KB DOC)

Figure S8 Excess H3 Causes Chromosome Segregation Defects (48 KB DOC)

Figure S9 Genomic Localization of *R.int-cnt1:ura4* ^+^ (R) (45 KB DOC)

Table S1 List of Strains Used in This Study (45 KB DOC)

Table S2 List of Primers Used in This Study (31 KB DOC)

## Abbreviations

ChIP: chromatin immunoprecipitation
FOA: 5-fluoro-orotic-acid
ROI: region of interest
RT: reverse transcriptase
RNAi: RNA interference
SPB: spindle pole body
