# Supplementary material for: Plasticity of Fission Yeast CENP-A Chromatin Driven by Relative Levels of Histone H3 and H4
Source: PLoS Genet. 2007 Jul 27;3(7):e121. doi: 10.1371/journal.pgen.0030121 (PMC1934396; doi:10.1371/journal.pgen.0030121)
Supplement: Figure S1 — (247 KB DOC) [file pgen.0030121.sg001.doc]

**A**

**ChIP: anti-Cnp1**


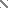


***otr1*R**


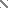


***otr1*L**

***cnt1***

***imr1*L**

***imr1*R**


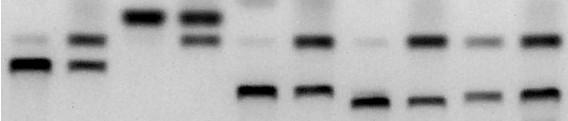


T

IP

T

IP

T

IP

T

IP

T

IP

T

IP

*— fbp*

1.4

32.5

47.4

11.1

26.7

8.2

*a*

*b*

*c*

*d*

*e*

*f*


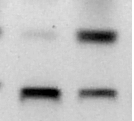


**B**


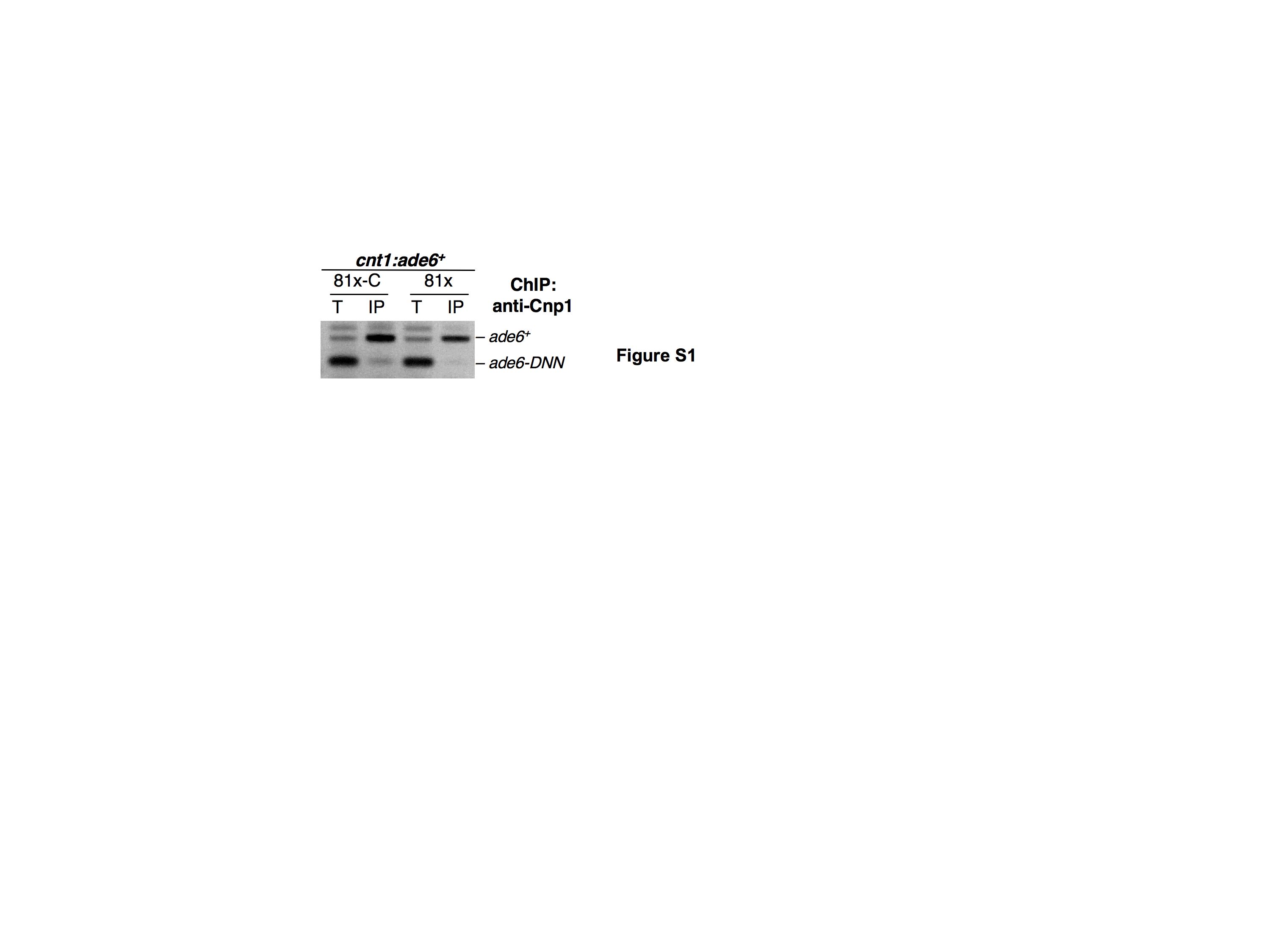


Figure S1: CENP-A^Cnp1^ chromatin is confined to the central domain and can associate with non-centromeric DNA

**A.** The distribution of endogenous CENP-A^Cnp1^ across *cen1* was assessed using anti-CENP-A^Cnp1^ antiserum for chromatin immunoprecipitation (ChIP). Regions *a* to *f* across cen1 were amplified by PCR from the input (T) and immunoprecipitated (IP) chromatin. Primers for regions *e-f* amplify a product from both *cnt1* and *cnt3*. Region *d* is specific to the left boundary of *cnt1* and *imr1*, regions *b* and *c* are located in imr1 (L/R) and region *a* is in the outer repeats. In all cases the euchromatic *fbp1* locus served as a negative control to assess the relative enrichment. These analyses show that endogenous CENP-A^Cnp1^ associates with the central domain (*cnt1* and *imr1)*, but not with flanking outer repeats. (FY972)

**B.** CENP-A^Cnp1^ associates with *ade6^+^* DNA inserted in the central domain of *cen1*.

ChIP analysis of CENP-A^Cnp1^ association with *cnt1:ade6*^+^ in wild-type cells overexpressing CENP-A^Cnp1^ or nothing from prep81x. DNA from the full length *ade6^+^* gene inserted at the *Nco*I site in the middle of *cnt1* is clearly enriched relative to the smaller PCR product from *ade6-DN/N* allele (missing 153 bp from the ORF). (FY378)
