# Supplementary material for: Plasticity of Fission Yeast CENP-A Chromatin Driven by Relative Levels of Histone H3 and H4
Source: PLoS Genet. 2007 Jul 27;3(7):e121. doi: 10.1371/journal.pgen.0030121 (PMC1934396; doi:10.1371/journal.pgen.0030121)
Supplement: Figure S2 — (338 KB DOC) [file pgen.0030121.sg002.doc]

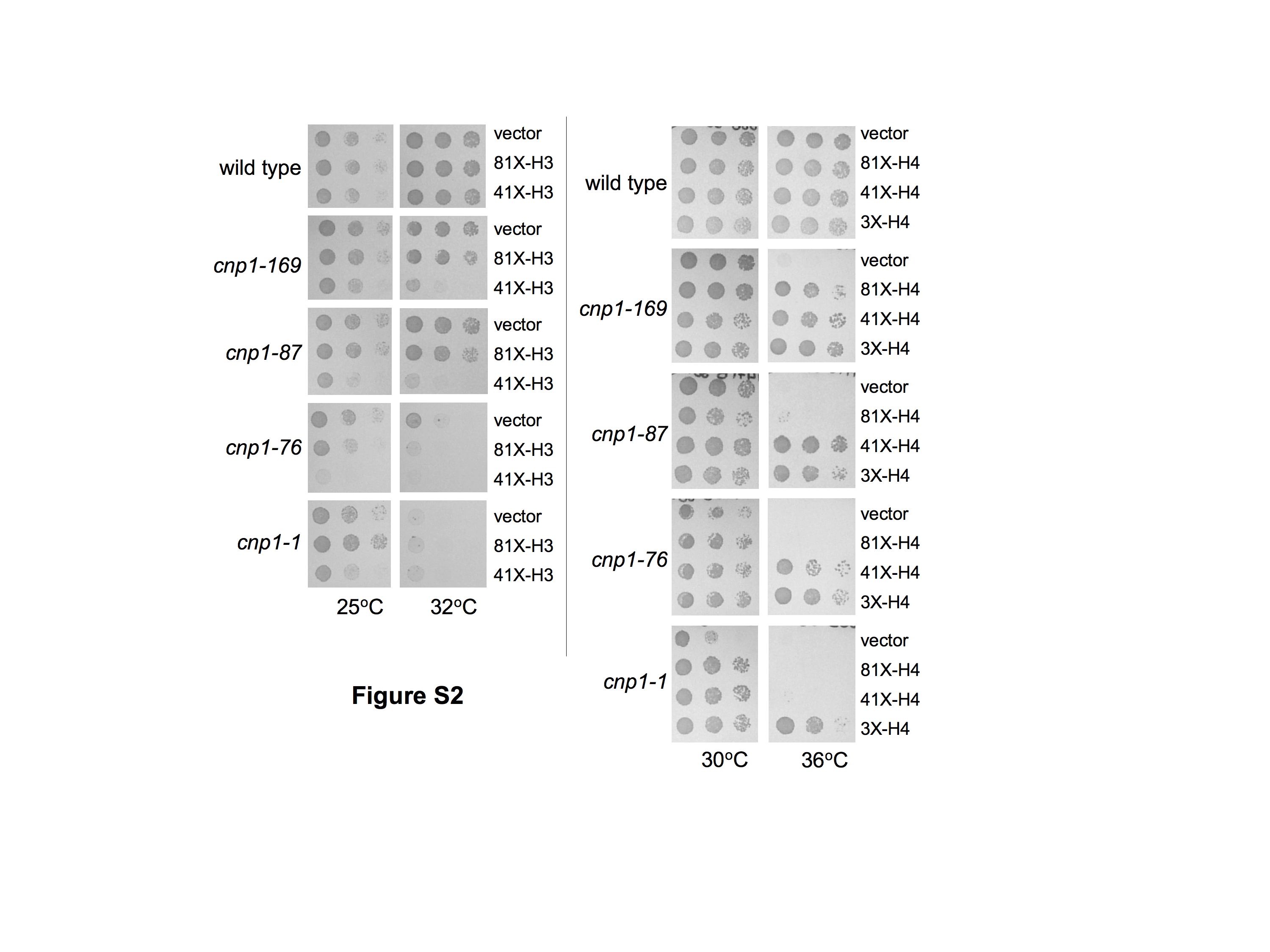


Figure S2: Correlation between strength of *cnp1* alleles (severity of temperature sensitive phenotype) and sensitivity to, or suppression by, different levels of H3 and H4, respectively.

(Left) Serial dilution assay of wild-type and temperature sensitive *cnp1-169*, *cnp1-87*, *cnp1-76*, or *cnp1-1* cells expressing additional histone H3 at low (prep81x-H3) and medium (prep41xH3) levels, compared to empty vector, on rich medium at 25^o^C and 32^o^C. (FY3033, FY4516, FY4471, FY4463, FY6960)

(Right) Serial dilution assay of wild-type and temperature sensitive *cnp1-169*, *cnp1-87*, *cnp1-76*, or *cnp1-1* cells expressing additional histone H4 at low (prep81x-H4), medium (prep41xH4), and high (prep3xH4) levels, compared to empty vector, on rich medium at 30^o^C and 36^o^C. (FY3033, FY4516, FY4471, FY4463, FY6960)
