# Supplementary material for: Plasticity of Fission Yeast CENP-A Chromatin Driven by Relative Levels of Histone H3 and H4
Source: PLoS Genet. 2007 Jul 27;3(7):e121. doi: 10.1371/journal.pgen.0030121 (PMC1934396; doi:10.1371/journal.pgen.0030121)
Supplement: Figure S3 — (1.4 MB DOC) [file pgen.0030121.sg003.doc]

14 kD 

21 kD 

30 kD 

14 kD 

21 kD 

30 kD 

14 kD 

21 kD 

30 kD 

Figure S3: CENP-A^Cnp1^ and histone H3 can be detected when overexpressed from prep3x. Anti-H3-Cterm does not cross react with CENP-A^Cnp1^.

Western analyses of extracts from wildtype cells overexpressing histone H3 or CENP-A^Cnp1^ from prep81x, prep41x and prep3x to give low, medium and high levels expression compared with cells containing empty vector prep3x.

Top membrane was incubated with anti- CENP-A^Cnp1^. Lower membranes were incubated with anti-Histone H3 recognizing the C terminus.

Higher levels of CENP-A^Cnp1^ and H3 clearly accumulate in cells expressing CENP-A^Cnp1^ or H3 from prep3x. No CENP-A^Cnp1^ signal is detected when this extract when incubated with anti-H3 C-term.
