# Supplementary material for: Plasticity of Fission Yeast CENP-A Chromatin Driven by Relative Levels of Histone H3 and H4
Source: PLoS Genet. 2007 Jul 27;3(7):e121. doi: 10.1371/journal.pgen.0030121 (PMC1934396; doi:10.1371/journal.pgen.0030121)
Supplement: Figure S4 — (191 KB DOC) [file pgen.0030121.sg004.doc]

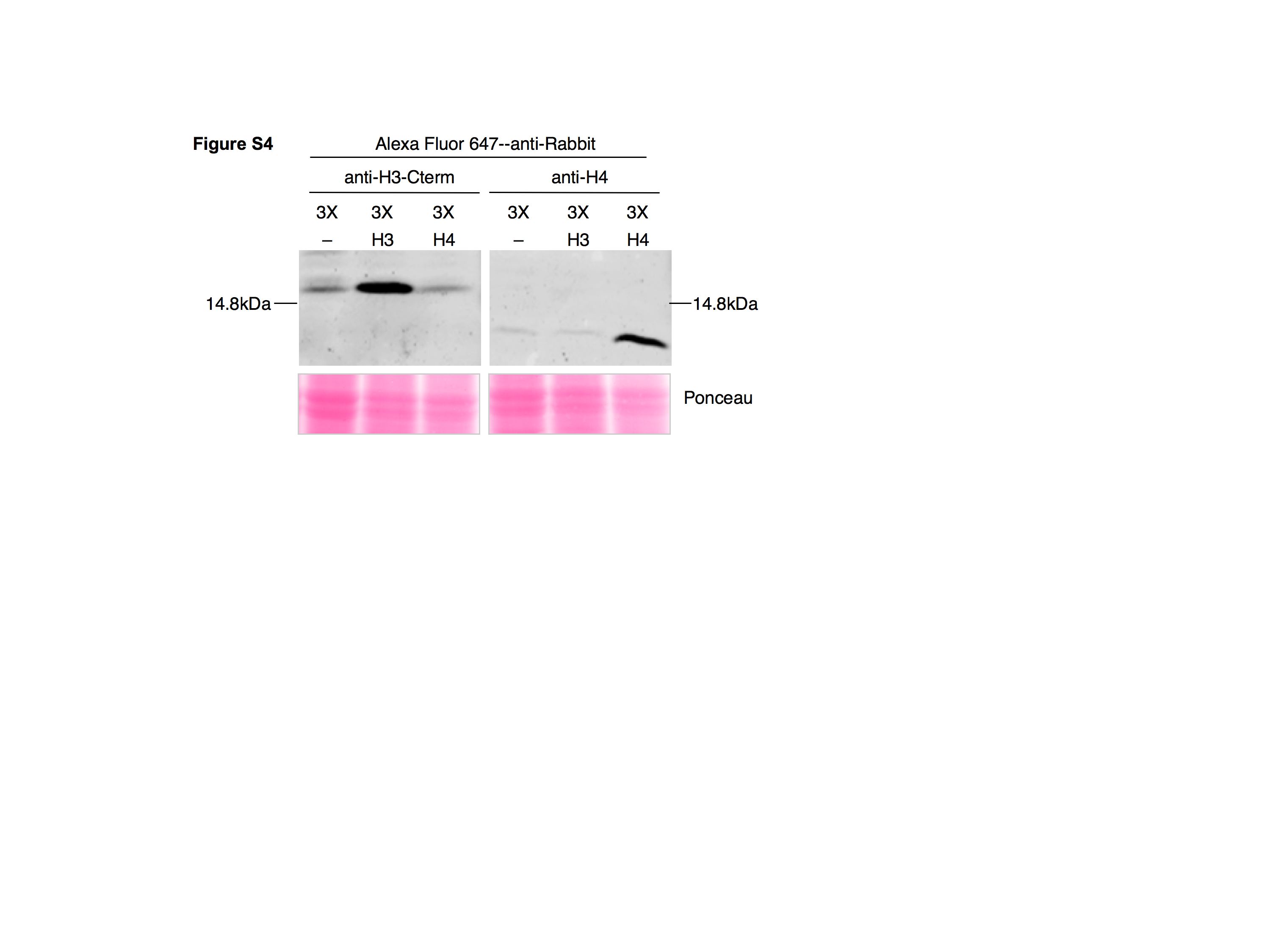


Figure S4: Total histone H3 and H4 levels are elevated 2.5 and 6 fold when overexpressed.

Western analysis was performed on extracts from cells expressing additional H3, H4 or nothing from the full *nmt1* promoter on prep3x. The resulting membranes were stained with Ponceau indicating that loading and transfer was relatively equivalent (Bottom). Membranes were incubated with anti-H3-Cterm (Abcam, 1791) or anti-H4 (Upstate, 05-858). Alexa Fluor 647 conjugated to Donkey anti-Rabbit-IgG (Invitrogen, A31573) was used to directly visualise the relative amount of histone H3 and H4. The levels of H3 and H4 fluorescent signal were captured directly on a Molecular Dynamics Storm PhosphorImager and quantified. H3 levels are 2.5 fold higher in cells expressing H3 from prep3x. H4 levels are 6 fold higher in cells expressing H4 from prep3x.
