# Supplementary material for: Plasticity of Fission Yeast CENP-A Chromatin Driven by Relative Levels of Histone H3 and H4
Source: PLoS Genet. 2007 Jul 27;3(7):e121. doi: 10.1371/journal.pgen.0030121 (PMC1934396; doi:10.1371/journal.pgen.0030121)
Supplement: Figure S5 — (378 KB DOC) [file pgen.0030121.sg005.doc]

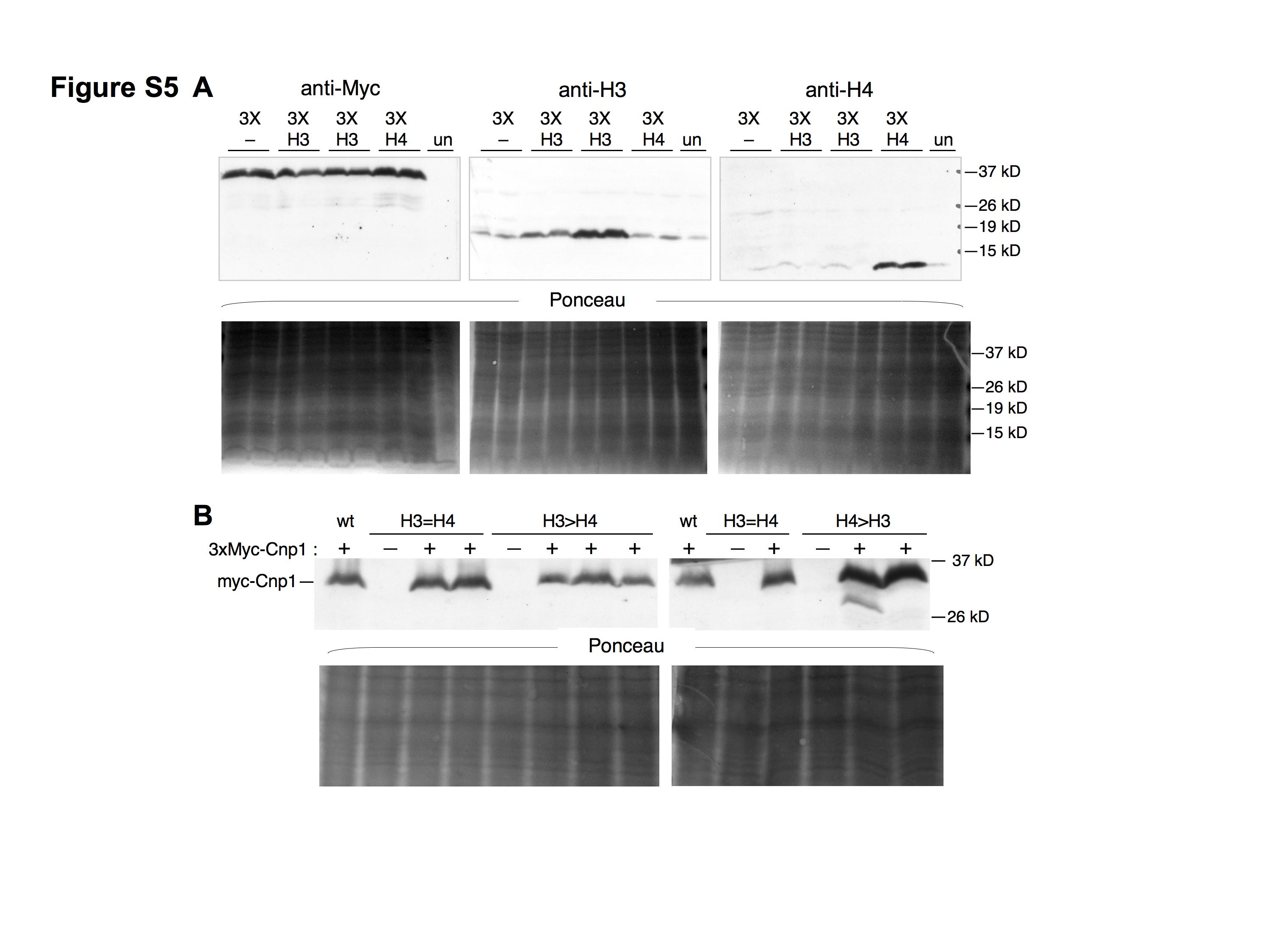


Figure S5: Levels of 13xMyc-CENP-A^Cnp1^ are unaffected by expression of excess histone H3 or H4.

Western analysis was performed on extracts from cells expressing additional H3, H4 or nothing from the full *nmt1* promoter on prep3x, or from H3=H4, H3>H4 or H3>H4 cells. All strains used expressed 13xMyc N-terminally tagged CENP-A^Cnp1^ (13xMyc-CENP-A^Cnp1^) at the *cnp1* locus from the *cnp1^+^* promoter as the only source of CENP-A^Cnp1^. The resulting membranes were incubated with anti-Myc to determine the relative levels of 13xMyc-CENP-A^Cnp1^. (FY1645, FY5925, FY5928, FY3569, FY8970, FY8971, FY8976, FY8977, FY8980, FY8981, FY8982)
