# Supplementary material for: Plasticity of Fission Yeast CENP-A Chromatin Driven by Relative Levels of Histone H3 and H4
Source: PLoS Genet. 2007 Jul 27;3(7):e121. doi: 10.1371/journal.pgen.0030121 (PMC1934396; doi:10.1371/journal.pgen.0030121)
Supplement: Figure S6 — (216 KB DOC) [file pgen.0030121.sg006.doc]

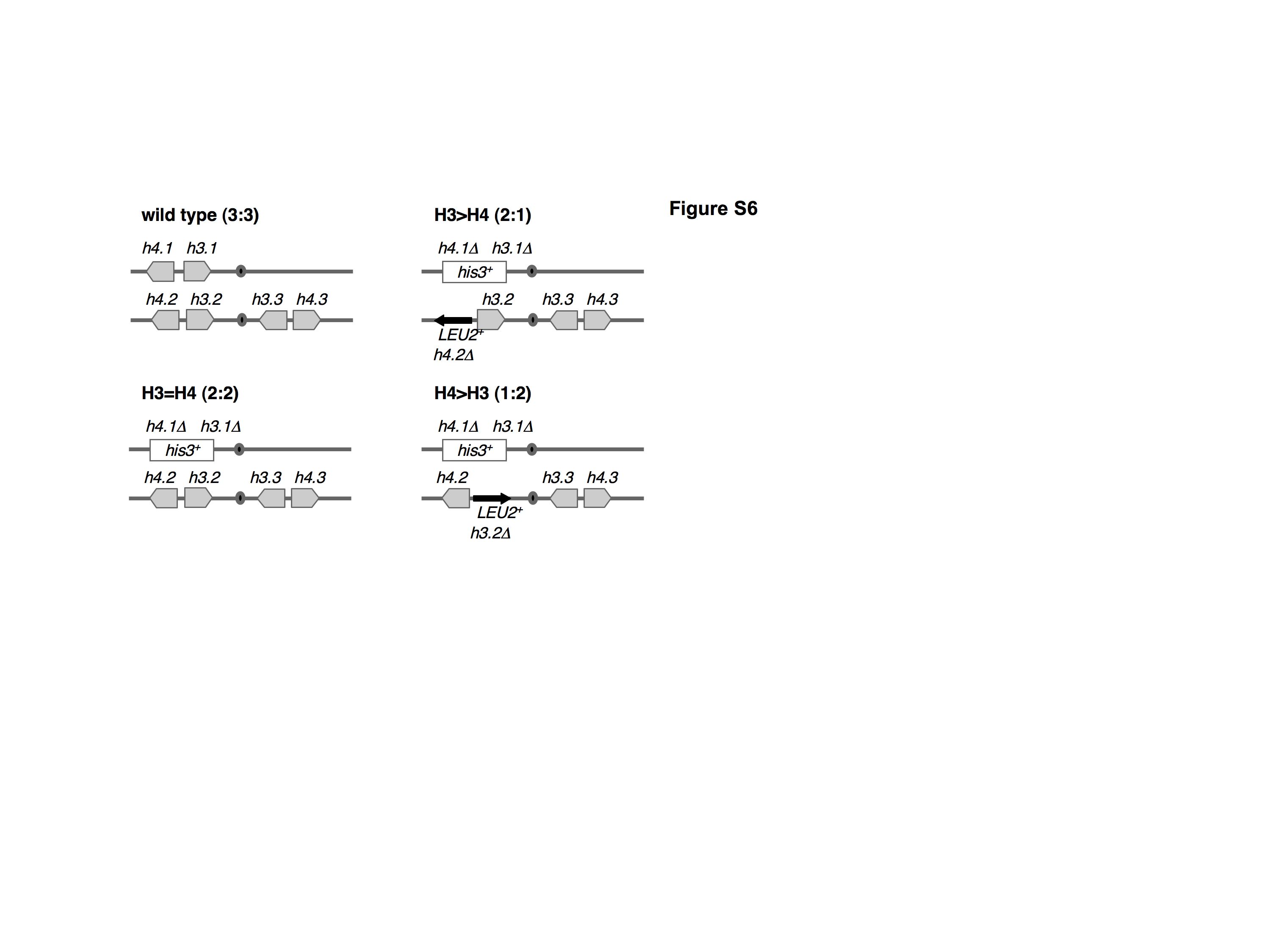


Figure S6: Construction of strains with altered H3:H4 gene ratios.

Wild type fission yeast contain three pairs of histone H3 and H4 genes arranged as divergently transcribed genes at three distinct locations in the genome giving a 3:3 ratio of H3:H4 genes. The histone *h3.1/h4.2* gene pair was deleted by replacement with the *his3^+^* marker gene creating a strain with a 2:2 ratio of H3:H4 genes (H3=H4). The histone individual *h3.2* or *h4.2* genes were subsequently replaced with the *LEU2^Sc^* marker gene (strains used in Figures 5 and 6) or *ura4*^+^ marker gene (strains used in Figure S7), creating strains with a H3:H4 imbalance; the H3>H4 strain has two histone H3 genes and only one H4 gene (H3:H4 2:1) while the H4>H3 strain has two histone H4 genes and only one H3 gene (H3:H4 1:2).
