# Supplementary material for: Plasticity of Fission Yeast CENP-A Chromatin Driven by Relative Levels of Histone H3 and H4
Source: PLoS Genet. 2007 Jul 27;3(7):e121. doi: 10.1371/journal.pgen.0030121 (PMC1934396; doi:10.1371/journal.pgen.0030121)
Supplement: Figure S7 — (49 KB DOC) [file pgen.0030121.sg007.doc]

**A**

| Mean intensity | H3=H4 | H4>H3 |
| --- | --- | --- |
| Sad1 spot | 887 (n=102) | 999 (n=132) |
| Sad1 background | 290 (n=130) | 359 (n=160) |
| Sad1 corrected | 512 | 640 |
| Cnp1 spot | 597 (n=102) | 872 (n=132) |
| Cnp1 background | 369 (n=130) | 494 (n=160) |
| Cnp1 corrected | 143 | 378 |
| Cnp1:Sad1 ratio | 0.24 | 0.59 |
| **Relative Cnp1 intensity** | **1** | **2.45** |

**B**

| Strain | Very bright | Bright | Faint | Undetectable | n |
| --- | --- | --- | --- | --- | --- |
| H3=H4 | 40 | 53 | 7 | 0 | 113 |
| H3>H4 | 2 | 7 | 54 | 37 | 89 |

Figure S7: Excess H3 causes delocalization of CENP-A^Cnp1^: quantification of CENP-A^Cnp1^ staining.

**A.** Comparison of CENP-A^Cnp1^ signal intensity in H3=H4 (2:2) versus H4>H3 (2:1), was performed using Metamorph software. Mean background intensity was calculated for each channel and subtracted from the mean of Sad1 or CENP-A^Cnp1^ spot intensities as appropriate. The mean corrected CENP-A^Cnp1^ intensity was divided by the mean corrected Sad1 intensity to normalise for efficiency of staining, giving a final value for CENP-A^Cnp1^ staining intensity. For the H3=H4 strain, 102 Sad1 and CENP-A^Cnp1^ spots were quantified (and 130 background measurements taken). For the H4>H3 strain, 132 Sad1 and CENP-A^Cnp1^ spots were quantified (and 160 background measurements taken). To determine the relative CENP-A^Cnp1^ staining intensity, the value for the H4>H3 strain was divided by the value for the H3=H4 strain. By this method, the CENP-A^Cnp1^ staining was calculated to be 2.5 fold brighter on average in the H4>H3 cells compared to the H3=H4 cells.

**B.** The method described in **A** was, however, determined not to be appropriate for measurement of relative CENP-A^Cnp1^ signal intensity in H3>H4 cells. This was because in many cells it was not possible to place the CENP-A^Cnp1^ ROI with confidence due to very low intensity or undetectable CENP-A^Cnp1^ staining at centromeres. Therefore, the CENP-A^Cnp1^ signals were simply categorised instead: ‘very bright’, ‘bright or ‘faint’ according to the maximum intensity within the spot. If no spot could be distinguished above background, it was recorded as ‘undetectable’.
