# Supplementary material for: Plasticity of Fission Yeast CENP-A Chromatin Driven by Relative Levels of Histone H3 and H4
Source: PLoS Genet. 2007 Jul 27;3(7):e121. doi: 10.1371/journal.pgen.0030121 (PMC1934396; doi:10.1371/journal.pgen.0030121)
Supplement: Figure S8 — (48 KB DOC) [file pgen.0030121.sg008.doc]

| Strain | Normal | CSD | Other | n |
| --- | --- | --- | --- | --- |
| H3=H4 (3:3) | 97.9 | 0.7 | 1.4 | 143 |
| H3=H4 (2:2) | 90.9 | 1.4 | 7.7 | 142 |
| H3>H4 (2:1) | 60.5 | 16.6 | 22.9 | 144 |
| H4>H3 (2:1) | 94.7 | 0 | 5.3 | 132 |

Figure S8: Excess H3 causes chromosome segregation defects

Anaphase cells were identified by presence of medium-long spindles and the segregation pattern was classified by inspection of the DAPI staining. CSD: chromosome segregation defects (includes lagging chromosomes and uneven segregation); Other: other unusual DNA/nuclei configurations.
