# Supplementary material for: Plasticity of Fission Yeast CENP-A Chromatin Driven by Relative Levels of Histone H3 and H4
Source: PLoS Genet. 2007 Jul 27;3(7):e121. doi: 10.1371/journal.pgen.0030121 (PMC1934396; doi:10.1371/journal.pgen.0030121)
Supplement: Figure S9 — (45 KB DOC) [file pgen.0030121.sg009.doc]

**A**

**Figure S9**

| **Cross** | **kan^R^** | ***ura4*** | **%RF** |
| --- | --- | --- | --- |
| R.Int x *cnp3::cnp3GFP-kanMX6* | 744 | 96 | 12.9% |
| R.Int x SPBC14F5.07::*kanMX6* | 570 | 25 | 4.39% |
| R.Int x rtt9::*kanMX6* | 1039 | 7 | 0.69% |

**B**

Figure S9: Genomic localization of *R.int-cnt1:ura4*^+^ (R)

**A.**  Genetic mapping. When the *R.int-cnt1:ura4*^+^ strain was crossed to *cnp3:GFP-kanMX6* strain (used in this study) it was found that the recombination frequency between the *kanMX6* and the *ura4*^+^ gene was 12.9% (RF= Recombination Frequency). Additional crosses using strains with the *kanMX6* gene replacing the ORFs SPBC14F5.07 and rtt109 (SPBC342.06c) 90 kb upstream or downstream *cnp3*^+^ indicated that *R.int-cnt1:ura4*^+^ (R) is genetically linked to both SPBC14F5.07 and rtt109 (SPBC342.06c).

**B.** Sequencing of the *R.int-cnt1:ura4*^+^ insertion. Using “Vectorette PCR” (Riley et al. 1990 Nucleic Acids Res. 18:2887-2890) we determined that *R.int-cnt1:ura4^+^* is inserted in an intragenic region between ORF SPBC342.01c encoding Alg6 (1.4 kb away from its ATG) and SPCB342.02 (0.3 kb away from its ATG). The length of the *cnt1* central core sequences from *cen1* flanking the *ura4*^+^ gene at *R.int-cnt1:ura4*^+^ was also determined using PCR and sequencing.
