# Supplementary material for: Plasticity of Fission Yeast CENP-A Chromatin Driven by Relative Levels of Histone H3 and H4
Source: PLoS Genet. 2007 Jul 27;3(7):e121. doi: 10.1371/journal.pgen.0030121 (PMC1934396; doi:10.1371/journal.pgen.0030121)
Supplement: Table S1 — (45 KB DOC) [file pgen.0030121.st001.doc]

**Table I. List of strains used in this study.**

Genotypes of strains used. For strains marked with an asterisk (*) only the relevant genotype is listed.

# Strain Genotype

__________

972 *h^-^*

1645 h+ *ade6-210 leu1-32 arg3-D4 his3-D1 ura4-D18*

4133 h+ *ade6-210 leu1-32 arg3-D4 his3-D1 ura4-DS/E*

4134 h- *ade6-210 leu1-32 arg3-D4 his3-D1 ura4-DS/E*

336 h- *ade6-210 leu1-32* *ura4-DS/E cnt1(Nco*I*):ura4*^+^ Allshire et al., 1995

534 h+ *ade6-210 leu1-32* *ura4-DS/E imr1*L(*Cla*I-*EcoR*I)*Hind*III:*ura4*^+^ oriI Allshire et al., 1995

525 h+ *ade6-210 leu1-32 ura4-DS/E* *imr1*L(*Nco*I-*Sph*I)*Hind*III:*ura4*^+^ oriI Allshire et al., 1995

496 h+ *ade6-210 leu1-32 ura4-DS/E imr1*L(dg-glu)*Nco*I:*ura4*^+^ oriI Allshire et al., 1995

965 h+ *ade6-210 leu1-32 ura4-DS/E otr1(dh-Nde*I-*Bgl*II)*Hind*III:*ura4*^+^ oriI Allshire et al., 1995

939 h+ *ade6-210 leu1-32 ura4-DS/E tRNAPhe-otr1(dh)Bgl*II:*ura4*^+^ oriII Allshire et al., 1995

4638 h+ *ade6-210 leu1-32* *ura4-DS/E cnt1(Nco*I*):ade6:ura4*^+^

6621 h- *ade6-210 leu1-32 ura4-DS/E R.int-cnt1(Nco*I*):ura4*^+^ Allshire et al., 1995

3033 *h^-^* *ade6-210 leu1-32 ura4-D18 arg3-D4 his3-D1 cnt1(Nco*I*):arg3^+^ cnt3(Nco*I*):ade6^+^*

*otr2(Hin*dIII*):ura4^+^ tel1*L*:his3^+^* Pidoux et al., 2003

4759 h *sim2-76 ade6-210 leu1-32 ura4-D18* (*) Pidoux et al., 2003

4761 h+ *sim2-87 ade6-210 leu1-32 ura4-D18* (*) Pidoux et al., 2003

4763 h *sim2-169 ade6-210 leu1-32 ura4-D18* (*) Pidoux et al., 2003

4771 h- *sim2-76* *ade6-210 leu1-32 ura4-DS/E cnt1(Nco*I*):ura4*^+^ (*) Pidoux et al., 2003

4765 h- *sim2-87* *ade6-210 leu1-32 ura4-DS/E cnt1(Nco*I*):ura4*^+^ (*) Pidoux et al., 2003

4764 h- *sim2-169* *ade6-210 leu1-32 ura4-DS/E cnt1(Nco*I*):ura4*^+^ (*) Pidoux et al., 2003

4463 *h^+^* *sim2-76* *ade6-210 leu1-32 ura4-D18 arg3-D4 his3-D1 cnt1:arg3^+^ cnt3:ade6^+^ otr2:ura4^+^ tel1*L*:his3^+^*

4471 h *sim2-87* *ade6-210 leu1-32 ura4-D18 arg3-D4 his3-D1 cnt1:arg3^+^ cnt3:ade6^+^ otr2:ura4^+^ tel1*L*:his3^+^*

4516 h- *sim2-169* *ade6-210 leu1-32 ura4-D18 arg3-D4 his3-D1 cnt1:arg3^+^ cnt3:ade6^+^ otr2:ura4^+^ tel1*L*:his3^+^*

6960 h- leu1-32 ura4- cnp1::ura4+ lys1+::cnp1-1 Takahashi et al., 2000

3569 h+ *h3.1/h4.1*::*his3*^+^ *ade6-210 arg3-D4 his3-D1 leu1-32 ura4-D18* Mellone et al., 2003

4813 h- *h3.2::LEU2^Sc^* *leu1-32 ura4-D18 ade6-210 his3-D1 arg3-D4* Mellone et al., 2003

4816 h+ *h4.2::LEU2^Sc^* *leu1-32 ura4-D18 ade6-210 his3-D1 arg3-D4* Mellone et al., 2003

4753 h- *h3.2::ura4^+^* *leu1-32 ura4-D18 ade6-210 his3-D1 arg3-D4* Mellone et al., 2003

4755 h- *h4.2::ura4^+^* *leu1-32 ura4-D18 ade6-210 his3-D1 arg3-D4* Mellone et al., 2003

7370 h- *h3.1/h4.1*::*his3*^+^ *h3.2::LEU2^Sc^* *ade6-210 arg3-D4 his3-D1 leu1-32 ura4-D18*

7372 h+ *h3.1/h4.1*::*his3*^+^ *h4.2::LEU2^Sc^* *ade6-210 arg3-D4 his3-D1 leu1-32 ura4-D18*

7448 h *h3.1/h4.1*::*his3*^+^ *h3.2::ura4^+^* *ade6-210 arg3-D4 his3-D1 leu1-32 ura4-D18*

7450 h *h3.1/h4.1*::*his3*^+^ *h4.2::ura4^+^* *ade6-210 arg3-D4 his3-D1 leu1-32 ura4-D18*

7980 h *h3.1/h4.1*::*his3*^+^ *ade6-210 arg3-D4 his3-D1 leu1-32 ura4-DS/E cnt1(Nco*I*):ura4*^+^

7982 h *h3.1/h4.1*::*his3*^+^ *ade6-210 arg3-D4 his3-D1 leu1-32 ura4-DS/E cnt1(Nco*I*):ade6:ura4*^+^

7984 h *h3.1/h4.1*::*his3*^+^ *ade6-210 arg3-D4 his3-D1 leu1-32 ura4-DS/E R.int-cnt1(Nco*I*):ura4*^+^

7390 h *h3.1/h4.1*::*his3*^+^ *h3.2::LEU2^Sc^* *ade6-210 his3-D1 leu1-32 ura4-DS/E cnt1(Nco*I*):ura4*^+^(*)

7986 h *h3.1/h4.1*::*his3*^+^ *h3.2::LEU2^Sc^* *ade6-210 his3-D1 leu1-32 ura4-DS/E cnt1(Nco*I*):ade6:ura4*^+^ (*)

7988 h *h3.1/h4.1*::*his3*^+^ *h3.2::LEU2^Sc^* *ade6-210 his3-D1 leu1-32 ura4-DS/E R.int-cnt1(Nco*I*):ura4*^+^ (*)

7398 h *h3.1/h4.1*::*his3*^+^ *h4.2::LEU2^Sc^* *ade6-210 his3-D1 leu1-32 ura4-DS/E cnt1(Nco*I*):ura4*^+^ (*)

7990 h *h3.1/h4.1*::*his3*^+^ *h4.2::LEU2^Sc^* *ade6-210 his3-D1 leu1-32 ura4-DS/E cnt1(Nco*I*):ade6:ura4*^+^ (*)

7993 h *h3.1/h4.1*::*his3*^+^ *h4.2::LEU2^Sc^* *ade6-210 his3-D1 leu1-32 ura4-DS/ E R.int-cnt1(Nco*I*):ura4*^+^ (*)

4812 h+ *mal2::mal2GFP-kanMX6* *ade6-210 leu1-32 ura4-D6* Jin et al., 2002

7946 h *mal2::mal2GFP-kanMX6* *ade6-210 leu1-32 ura4-DS/E cnt1(Nco*I*):ura4*^+^ (*)

7948 h *mal2::mal2GFP-kanMX6* *ade6-210 leu1-32 ura4-DS/E R.int-cnt1(Nco*I*):ura4*^+^ (*)

7950 h *mal2::mal2GFP-kanMX6* *ade6-210 leu1-32 ura4-DS/E cnt1(Nco*I*):ade6:ura4*^+^ (*)

5145 h+ *cnp3::cnp3GFP-kanMX6* *ade6-210 leu1-32 arg3-D4 his3-D1 ura4-D18*

*7952* h *cnp3::cnp3GFP-kanMX6* *ade6-210 leu1-32 ura4-DS/E cnt1(Nco*I*):ura4*^+^ (*)

7954 h *cnp3::cnp3GFP-kanMX6* *ade6-210 leu1-32 ura4-DS/E R.int-cnt1(Nco*I*):ura4*^+^ (*)

*7956* h *cnp3::cnp3GFP-kanMX6* *ade6-210 leu1-32 ura4-DS/E cnt1(Nco*I*):ade6:ura4*^+^ (*)

5201 h+ *sim4::sim4GFP-kanMX6* *ade6-210 leu1-32 arg3-D4 his3-D1 ura4-D18* Pidoux et al., 2003

*7958* h *sim4::sim4GFP-kanMX6* *ade6-210 leu1-32 ura4-DS/E cnt1(Nco*I*):ura4*^+^ (*)

7960 h *sim4::sim4GFP-kanMX6* *ade6-210 leu1-32 ura4-DS/E R.int-cnt1(Nco*I*):ura4*^+^ (*)

*7962* h *sim4::sim4GFP-kanMX6* *ade6-210 leu1-32 ura4-DS/E cnt1(Nco*I*):ade6:ura4*^+^ (*)

5925 h+ *cnp1::13MYC-cnp1 ade6-210 leu1-32 his3-D1* *ura4-D18* (*)

5928 h- *cnp1::13MYC-cnp1 ade6-210 leu1-32 his3-D1* *ura4-D18* (*)

8970 h *cnp1::13MYC-cnp1 h3.1/h4.1*::*his3*^+^ *ade6-210 arg3-D4 his3-D1 leu1-32 ura4-D18* (*)

8971 h *cnp1::13MYC-cnp1 h3.1/h4.1*::*his3*^+^ *ade6-210 arg3-D4 his3-D1 leu1-32 ura4-D18* (*)

8976 h *cnp1::13MYC-cnp1 h3.1/h4.1*::*his3*^+^ *h3.2::ura4^+^* *ade6-210 his3-D1 leu1-32 ura4-D18* (*)

8977 h *cnp1::13MYC-cnp1 h3.1/h4.1*::*his3*^+^ *h3.2::ura4^+^* *ade6-210 his3-D1 leu1-32 ura4-D18* (*)

8980 h *cnp1::13MYC-cnp1 h3.1/h4.1*::*his3*^+^ *h4.2::ura4^+^* *ade6-210 his3-D1 leu1-32 ura4-D18* (*)

8981 h *cnp1::13MYC-cnp1 h3.1/h4.1*::*his3*^+^ *h4.2::ura4^+^* *ade6-210 his3-D1 leu1-32 ura4-D18* (*)

8982 h *cnp1::13MYC-cnp1 h3.1/h4.1*::*his3*^+^ *h4.2::ura4^+^* *ade6-210 his3-D1 leu1-32 ura4-D18* (*)

**Figure**

1A FY939, FY965, FY496, FY525, FY534, FY336, FY6621

1B, C FY336, FY4638, FY6621

1D FY939, FY336, FY4638, FY6621, FY4133

1E FY972, FY939, FY336, FY4638, FY6621

2A FY336, FY4771, FY4765, FY4764, FY972, FY1645

2B FY1645, FY4759, FY4761, FY4763, FY6960

2C FY336, FY4638, FY6621

2D, E FY3033, FY4471

3A FY336, FY4638, FY6621

3B, C FY336, FY4638

4A FY336, FY4638, FY6621

4B, C FY336, FY4638

5A, B FY336, FY7980, FY7398, FY7390, FY4638, FY7982, FY7990, FY7986, FY6621, FY7984, FY7993,FY7988

5C, D FY7980, FY7398, FY7390, FY7982, FY7990, FY7986

5E FY3569, FY7450

6A, B FY3569, FY7370, FY7372, FY7448, FY7450

7A, B FY7946, FY7959, FY7948, FY7952, FY7956, FY7954, FY7958, FY7962, FY7960
