# Supplementary material for: Plasticity of Fission Yeast CENP-A Chromatin Driven by Relative Levels of Histone H3 and H4
Source: PLoS Genet. 2007 Jul 27;3(7):e121. doi: 10.1371/journal.pgen.0030121 (PMC1934396; doi:10.1371/journal.pgen.0030121)
Supplement: Table S2 — (31 KB DOC) [file pgen.0030121.st002.doc]

**Table II. List of primers used in this study.**

# Primers

WA26 AACAATAAACACGAATGCCTC

WA27 ATAGTACCATGCGATTGTCTG

WA28 GGCTACCAGCATTGTTATTCATAACC

WA29 GGATATATGTATTCTTGCACTC

WA31 CACATCATCGTCGTACTACAT

WA32 GATATCATCTATATTTAATGACTACT

WA33 AATGACAATTCCCCACTAGCC

WA34 ACTTCAGCTAGGATTCACCTGG

WA41 GAGGGGATGAAAAATCCCAT

WA42 TTCGACAACAGGATTACGACC

imrEf GCTTTATCATAGTATTTTAGGC

imrEr AATACTTGGTATGTTAAAGTGG

imrCf ATAGTTATTGAAAGGCTGCG

imrCr CGACTTTGTACTTTGTCCAG

imrC2f TTGAAAGAGCTTGACACGTTTT

imrC2r TTCCTGCTGAGGCTAAGTATCTG

F7-cnt1 CAACTTACATCAGCATACTGG

R9-cnt1 TGATCCTTTTGTTACCGGCG

F10-cnt1 TATTCCTTAACTCATCATGC

R12-cnt1 GCTTGCTCTTTTCGTTCCTG

Cen-REV CGTCTTGTAGCTGCATGTGAA

Cen-FOR GAAAACACATCGTTGTCTTCAGAG

H3.2-5-XhoI-RI TACTACCTCGAGGAATTCATGGCTCGTACCAAGCAAACTGC

H3.2-3-Xho-Bam TACTACCTCGAGGGATCCTTAAGAGCGTTCGCCACGAAG

H4.2-5-XhoI-RI TACTACCTCGAGGAATTCATGTCTGGTCGTGGAAAAGG

H4.2-3-Xho-Bam TACTACCTCGAGGGATCCTTAACCACCGAAACCATAAATGG

cnp1-5-XhoI TACTACCTCGAGatggcaaagaaatctttaatggc

cnp1-3-Xho-Bam TACTACCTCGAGGGATCCTCAAGCACCACGAATCCTCCTGG

M32 CGGCATCTCTGCACATGTCGTGTTTTCTTACCGTATTGTCCTACCAAGAA

CCCTCGAGGAGAACTTCTAGTATATCC

M35 GTGGTAATGTTGTAGGAGCATGTTTAATAAATTACTATAGCAAATTACCGT

CGTAAGGCC GTT TCTG
